# A Subset of Replication Proteins Enhances Origin Recognition and Lytic Replication by the Epstein-Barr Virus ZEBRA Protein

**DOI:** 10.1371/journal.ppat.1001054

**Published:** 2010-08-19

**Authors:** Ayman El-Guindy, Lee Heston, George Miller

**Affiliations:** 1 Department of Molecular Biophysics and Biochemistry, Yale University School of Medicine, New Haven, Connecticut, United States of America; 2 Department of Pediatrics, Yale University School of Medicine, New Haven, Connecticut, United States of America; 3 Department of Epidemiology and Public Health, Yale University School of Medicine, New Haven, Connecticut, United States of America; University of Wisconsin-Madison, United States of America

## Abstract

ZEBRA is a site-specific DNA binding protein that functions as a transcriptional activator and as an origin binding protein. Both activities require that ZEBRA recognizes DNA motifs that are scattered along the viral genome. The mechanism by which ZEBRA discriminates between the origin of lytic replication and promoters of EBV early genes is not well understood. We explored the hypothesis that activation of replication requires stronger association between ZEBRA and DNA than does transcription. A ZEBRA mutant, Z(S173A), at a phosphorylation site and three point mutants in the DNA recognition domain of ZEBRA, namely Z(Y180E), Z(R187K) and Z(K188A), were similarly deficient at activating lytic DNA replication and expression of late gene expression but were competent to activate transcription of viral early lytic genes. These mutants all exhibited reduced capacity to interact with DNA as assessed by EMSA, ChIP and an *in vivo* biotinylated DNA pull-down assay. Over-expression of three virally encoded replication proteins, namely the primase (BSLF1), the single-stranded DNA-binding protein (BALF2) and the DNA polymerase processivity factor (BMRF1), partially rescued the replication defect in these mutants and enhanced ZEBRA's interaction with oriLyt. The findings demonstrate a functional role of replication proteins in stabilizing the association of ZEBRA with viral DNA. Enhanced binding of ZEBRA to oriLyt is crucial for lytic viral DNA replication.

## Author Summary

Epstein-Barr virus encodes a protein, ZEBRA, which plays an essential role in the switch between viral latency and the viral lytic cycle. ZEBRA activates transcription of early viral genes and also promotes lytic viral DNA replication. It is not understood how these two functions are discriminated. We studied five ZEBRA mutants that are impaired in activation of replication but are wild-type in the capacity to induce transcription of early viral genes. We demonstrate that these five mutants are impaired in binding to viral DNA regulatory sites. Therefore, replication required stronger interactions between ZEBRA and viral DNA than did transcription. Three components of the EBV-encoded replication machinery, including the single-stranded DNA binding protein, the polymerase processivity factor and the primase markedly enhanced the interaction of ZEBRA with viral DNA. These three components partially rescued the defect in ZEBRA mutants that were impaired in replication. The results suggest that through protein-protein interaction, replication proteins play a role in enhancing ZEBRA's association with the origin of DNA replication and other regulatory sites.

## Introduction

There are many gaps in our understanding of the process by which the Epstein-Barr virus (EBV) lytic replication machinery assemble on DNA sites present in the viral genome.

EBV encodes an essential bZIP protein known as ZEBRA (aka Zta, Z and BZLF1) that functions as a transcription activator of viral and cellular genes and as an origin binding protein during lytic DNA replication. An EB viral genome that lacks the open reading frame encoding ZEBRA, *bzlf1*, loses its ability to activate lytic gene expression and DNA replication [1]. ZEBRA interacts both with promoters and with origins of lytic replication through DNA sequences known as ZEBRA response elements (ZREs) that are common to both types of DNA regulatory regions [2], [3], [4]. It is unknown how ZEBRA distinguishes between a replication site and a transcription activation site. The mechanism by which ZEBRA activates transcription relies on its capacity to bind DNA and to form physical contact with a number of cellular proteins. ZEBRA binds to a wide variety of ZREs located in target promoters. Some of these response elements contain methylated CpG motifs to which ZEBRA binds with high preference [5]. The protein also forms stable transcriptional initiation complexes with basic components of the transcription machinery such as TBP, TFIID, and the transcription co-activator CBP [6], [7], [8]. Since ZEBRA augments the histone acetyl transferase (HAT) activity of CBP, interaction of ZEBRA with CBP increases promoter accessibility [9].

Activation of viral DNA synthesis during the lytic phase of the EBV life cycle is dependent on the capacity of ZEBRA to efficiently recognize a large (∼1 kb) complex intergenic region that serves as the origin of replication. This region, known as oriLyt, consists of essential and auxiliary segments [10]. The two essential components of oriLyt, the upstream and downstream elements, together constitute the minimal origin of DNA replication [2], [11], [12]. The auxiliary component serves as an enhancer element that augments DNA replication [13], [14]. ZEBRA recognizes the origin of lytic DNA replication (oriLyt) by interacting with seven ZEBRA-binding sites [12], [15]. Mutation of all seven binding motifs in the background of a recombinant virus drastically reduces production of infectious virus particles [16]. These ZEBRA binding elements are located in two non-contiguous regions of oriLyt. Four elements are present in the upstream core region of oriLyt and overlap with the promoter of the BHLF1 open reading frame [3]. Knocking out any of these four elements was deleterious for amplification of an oriLyt-containing plasmid in a transient replication assay [17]. Three additional ZEBRA binding elements located mainly in the enhancer region are dispensable for viral replication [17].

The current model for the role of ZEBRA in lytic DNA replication suggests that the protein serves as a physical link between oriLyt and core components of the replication machinery [18], [19]. The six core replication factors encoded by EBV are the DNA polymerase (BALF5); the polymerase processivity factor (BMRF1); the helicase (BBLF4); the primase (BSLF1); the primase associated factor (BBLF2/3), and the single-stranded DNA binding protein (BALF2) [4]. Corroboration for the proposed role of ZEBRA in replication is inferred from data showing that ZEBRA interacts with almost all components of the viral replication machinery, with the exception of the single-stranded DNA binding protein (BALF2) [18], [20], [21], [22]. The function of tethering replication proteins to oriLyt is not limited to ZEBRA; the transactivation domains of Sp1 and ZBP89 interact with BMRF1 and BALF5 and target them to the downstream region of oriLyt [18], [23]. Similarly, ZBRK1, a cellular DNA binding zinc finger protein, serves as a contact point for BBLF2/3 on oriLyt [19]. Deletion of the ZBRK1 binding site present in the downstream region of oriLyt reduced oriLyt-dependent replication of a transiently transfected plasmid. Binding of these cellular transcription factors is not essential but contributes to replication efficiency.

ZEBRA mutants that activate transcription but not replication are valuable in furthering our understanding of the process of EBV lytic DNA replication. ZEBRA is phosphorylated *in vivo* at multiple sites [24]. Phosphorylation of ZEBRA at S173 regulates lytic viral replication [25]. Serine 173 is located in a region N-terminal to the DNA binding domain of ZEBRA. This region, known as the regulatory domain, regulates the DNA binding activity of the protein [25], [26], [27]. Alanine substitution of the phosphoacceptor site S173 reduced the capacity of ZEBRA to bind to DNA *in vitro* and *in vivo* [25]. Attenuation in DNA binding correlated with a defect in the capacity of ZEBRA to stimulate lytic viral replication. However, it had no effect on the ability of ZEBRA to activate transcription of downstream viral target genes. Thus phosphorylation of S173 segregates the two main functions of ZEBRA, namely activation of transcription and activation of viral replication. In addition, the S173A mutant demonstrates that activation of transcription is not sufficient to stimulate viral replication. Additional proof for the role of phosphorylation of S173 in replication was attained when a phosphomimetic substitution mutant Z(S173D) activated both transcription and replication and was competent to bind DNA to the same extent as wild-type (wt) ZEBRA. Therefore, phosphorylation of ZEBRA at S173 functionally mimics ATP binding in other origin binding proteins by enhancing the DNA binding activity of ZEBRA to all ZREs in general and not to a specific site [25].

In a comprehensive mutagenesis study of the DNA binding domain of ZEBRA we identified ZEBRA mutants that arrested the EBV lytic cycle at different stages [28]. Two of these mutants, Z(Y180E) and Z(K188A), caused lytic cycle arrest prior to viral replication. They reproducibly activated expression of viral early genes but were defective in inducing amplification of EBV DNA and late gene expression [28]. These mutants did not affect the phosphorylation site in the regulatory domain, S173, but changed specific residues within the DNA recognition domain. The availability of replication defective (RD) ZEBRA mutants prompted us to investigate the effect of alterations in the DNA binding activity of ZEBRA on viral replication. If replication is indeed less tolerant than transcription for weak interaction between ZEBRA and DNA, then stronger association with oriLyt is necessary and might play a critical role in origin activation. Augmentation of ZEBRA binding to oriLyt is likely to be mediated by factors specific for viral replication. For example in budding yeast, interaction of the ORC with Cdc6 enhances its interaction with the origin of replication [29]. Here we describe a new role for three components of the EBV replication complex, namely, the primase, the single-stranded DNA binding protein and the DNA processivity factor. We show that over-expression of these three replication proteins is sufficient to increase the association of ZEBRA with viral DNA. This augmentation in DNA binding suppressed the phenotype of ZEBRA replication defective mutants and partially restored viral genome amplification and late gene expression. Our findings represent the first indication that three replication proteins play a role in enhancing the interaction between ZEBRA and viral DNA thereby promoting origin recognition, a process that is exquisitely sensitive to the DNA binding activity of ZEBRA.

## Results

### Characterization of ZEBRA replication defective (RD) mutants

**Figure 1:**
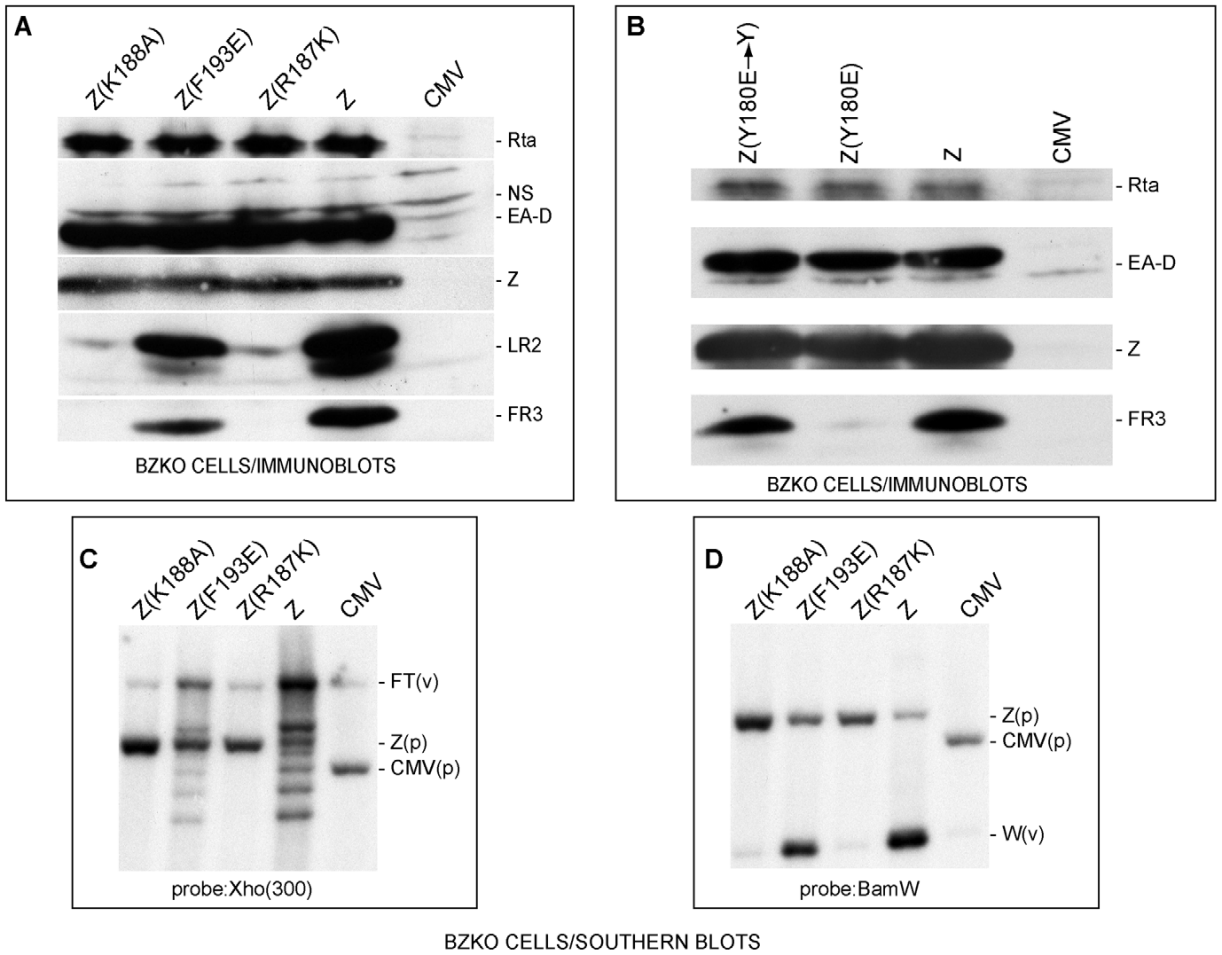
ZEBRA mutants defective in lytic replication and late gene expression. (A) Z(R187K) and Z(K188A) fail to activate two late proteins, FR3 and LR2. Immunoblot analysis of extracts prepared from BZKO cells transfected with expression vectors encoding wt ZEBRA (Z) and the following ZEBRA mutants: Z(K188A), Z(F193E), and Z(R187K). The membrane was probed for the EBV gene products, Rta, EA-D (early antigen-diffuse, the DNA polymerase processivity factor or BMRF1), ZEBRA, LR2 and FR3. NS, non specific. B) Z(Y180E), but not its revertant, specifically fails to activate EBV late gene expression. An immunoblot of transfected BZKO cells was probed with antibodies as described in panel A. C) and D) Southern blots assessing lytic viral DNA replication. Total DNA was extracted 48 hrs after transfection of the expression vectors. The DNA was digested with BamH1. The blots were probed with either a 0.3 kb subfragment of the Xho 1.9 fragment (Fig. 1C) or the BamH1 W fragment (Fig. 1D). FT, fused termini of the endogenous viral genome (v); Z(p), plasmid encoding ZEBRA; CMV, (p) empty plasmid; W(v), the BamW fragment of the endogenous virus (v).

Previously we described three ZEBRA mutants which activated expression of early genes but failed to activate viral replication and late gene expression. The ZEBRA mutants that reproducibly exhibited replication defective phenotype were: Z(S173A) in the regulatory domain and Z(Y180E) and Z(K188A) in the DNA recognition domain [25], [28]. In further exploration of this phenomenon we identified a fourth ZEBRA RD mutant with a conservative arginine to lysine substitution at position 187. Fig. 1A, C and D compare the phenotype of Z(R187K) to wt ZEBRA, Z(K188A) and Z(F193E). Z(K188A) served as a typical ZEBRA RD mutant; the mutant Z(F193E) was partially defective in induction of late genes and DNA replication. Expression of Z(R187K) in BZKO cells induced a pattern of lytic gene expression that mimicked Z(K188A); it fully activated expression of two early proteins, Rta and EA-D (aka BMRF1), encoded by *brlf1* and *bmrf1*, but failed to activate synthesis of two late proteins BFRF3 (FR3) (a component of the viral capsid) and BLRF2 (LR2) (a tegument protein) (Fig. 1A). Rta and EA-D are direct targets of ZEBRA; their expression is governed by the ability of ZEBRA to bind to their corresponding promoters, Rp and BMRF1p, respectively [30], [31], [32]. Activation of expression of the two late proteins, FR3 and LR2, is associated with the capacity of ZEBRA to induce lytic viral replication [33].

To demonstrate that the introduced point mutations were the sole cause for the observed defect in late gene expression, the mutant Z(Y180E) was reverted to its original amino acid composition, i.e. tyrosine. As expected, Z(Y180E) was impaired in activating late gene expression while the revertant mutant Z(Y180E→Y) was competent to activate late gene expression to the same level as wt ZEBRA (Fig. 1B).

To examine whether the defect in late gene expression was due to a failure in stimulating viral replication, we tested the capacity of Z(R187K) to induce viral genome amplification by probing for two different regions of viral DNA. First, we probed for a region upstream of the viral terminal repeats (TRs). During lytic viral replication linear viral genomes are synthesized. These linear forms differ in their number of terminal repeats and are detected on a Southern blot as a ladder [34]. In Fig. 1C, wt ZEBRA induced the formation of a replication ladder. Z(F193E) was slightly impaired and resulted in a less intense ladder than wt ZEBRA. The two late mutants Z(R187K) and Z(K188A) failed to induce the replication ladder. Comparable results were observed when a Southern blot of a parallel experiment was probed for the reiterated *BamH1* W sub-fragment of EBV DNA (Fig. 1D). All the RD mutants were defective at amplifying viral DNA when assessed by qPCR (Fig. S3). Based on these results and our previous studies, we conclude that RD mutants Z(S173A), Z(Y180E), Z(R187K), and Z(K188A) are competent to activate expression of early viral proteins but incompetent to activate lytic viral DNA replication and late gene expression (see also Fig. S3).

### ZEBRA RD mutants can activate transcription of the endogenous *brlf1* gene

**Figure 2:**
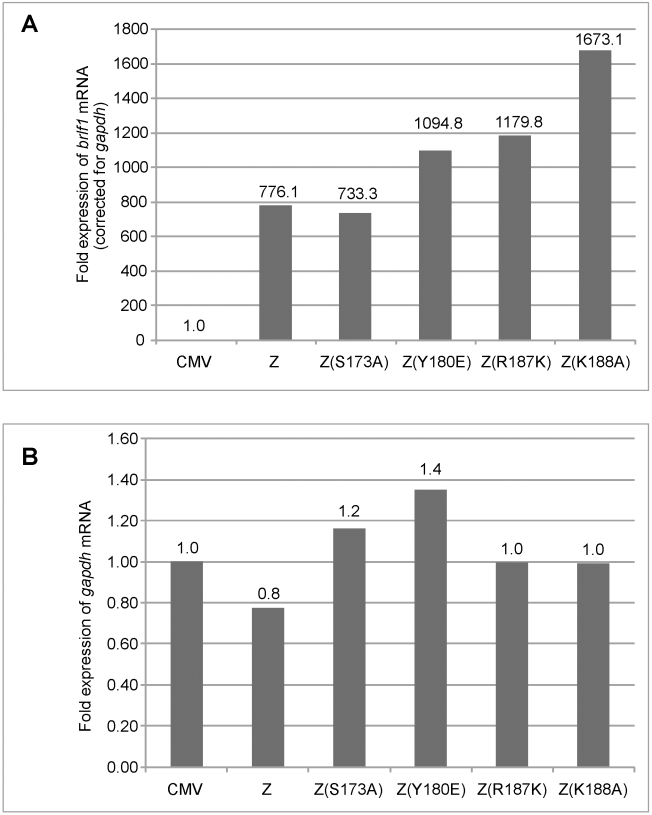
Replication defective ZEBRA mutants activate *brlf1* gene expression to wild-type levels. Total RNA was purified from BZKO cells 48 hrs after transfection with vector, wt ZEBRA or the indicated replication defective ZEBRA mutants. Expression of the *brlf1* (panel A) and the *gapdh* (panel B) messages was measured by quantitative RT-PCR and normalized to the level of these transcripts in BZKO cells transfected with empty vector. Fold expression of *brlf1* mRNA was corrected for the amount of *gapdh* transcripts detected in the same sample.

Although the data in Fig. 1 showed that the four replication defective mutants activated expression of two early proteins, Rta and EA-D to the same level as wt ZEBRA, this result did not directly assess the capacity of the mutants to activate transcription from early promoters. The level of *brlf1* transcripts is particularly important since activation of the *brlf1* promoter by direct binding of ZEBRA is a crucial initial event in activation of the EBV lytic cycle [5], [30], [31], [32], [35]. Therefore, using quantitative RT-PCR we measured the level of endogenous *brlf1* mRNA, encoding Rta, in BZKO cells expressing each of the four ZEBRA RD mutants. We found that wt ZEBRA induced expression of the *brlf1* message by 776-fold relative to the background level of *brlf1* mRNA detected in cells transfected with empty vector (Fig. 2A). The level of *brlf1* expression was corrected for the corresponding level of the *gapdh* transcript measured in each sample (Fig. 2B). The ZEBRA RD mutants, Z(S173A), Z(Y180E), Z(R187K) and Z(K188A), reproducibly activated expression of the *brlf1* message to levels similar or higher than that of wt ZEBRA (Fig. 2, S4A and S5). Therefore, despite a clear defect in the capacity of these ZEBRA mutants to activate viral replication, the mutants were fully competent to activate transcription of the early *brlf1* gene.

### The RD ZEBRA mutants activate transcription of EBV-encoded replication genes

**Figure 3:**
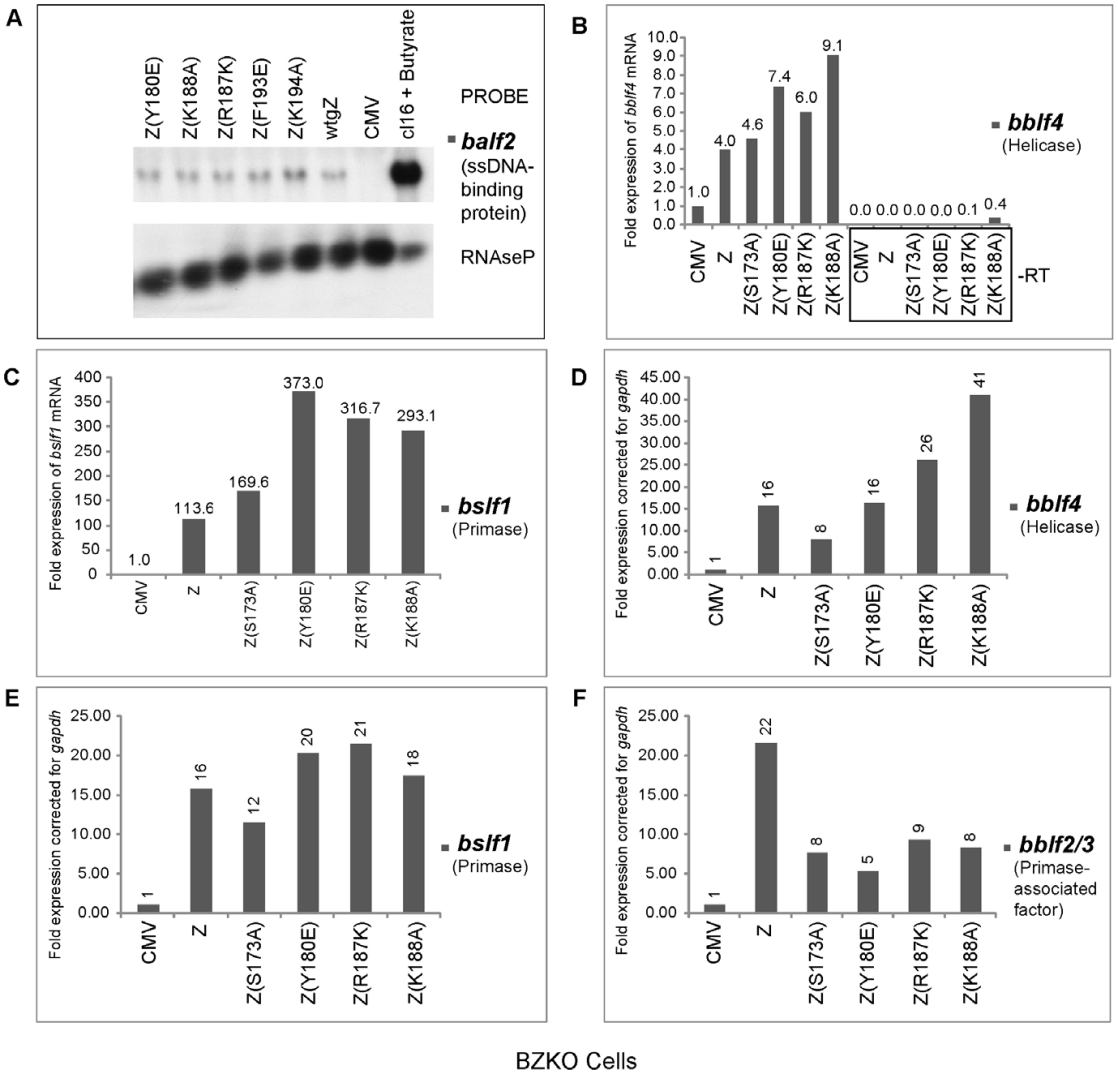
ZEBRA replication defective (RD) mutants are competent at activating expression of EBV genes encoding the viral lytic replication machinery. A) Northern blot analysis of *balf2* mRNA isolated from BZKO cells transfected with wild type BZLF1 and ZEBRA point mutants. Cells were harvested after 48 h. RNA prepared from HH514-16 (cl16) cells treated with sodium butyrate was used as a positive control for the expression of *balf2* mRNA. RNaseP served as a control for the total amount of cellular RNA loaded on the gel. Panels B, C, D, E and F represent quantitative RT-PCR to measure the expression level of the EBV helicase (*bblf4*) (Fig. 3B and D), the EBV primase (*bslf1*) (Fig. 3C and E) and the EBV primase-associated factor (*bblf2/3*) (Fig. 3F), in cells transfected with CMV (empty vector) or expression vectors for wt ZEBRA or mutant ZEBRA proteins. Fold expression for each transcript was calculated using the standard curve method and was corrected for the level of *gapdh* mRNA. In Figs. 3B and C, the reverse transcription reaction was performed using gene specific primers. In Fig. D, E and F, cDNA was synthesized using a mixture of poly(dT) and random hexamer primers.

In addition to its role in replication, expression of ZEBRA leads to activation of transcription of early lytic cycle genes, six of which constitute core components of the viral lytic replication machinery [4], [36]. The defect observed with the ZEBRA RD mutants could be attributed to failure to activate transcription of one or more genes encoding essential replication proteins. To investigate this possibility, we examined the capacity of the ZEBRA mutants to activate transcription of the different components of the viral replication machinery. Expression of *balf2*, the gene encoding the single-stranded DNA binding protein was examined by expressing five ZEBRA mutants in BZKO cells. Three of these mutants, Z(Y180E), Z(K188A) and Z(R187K), are markedly defective in activating late gene expression and viral replication, Fig. 1, Fig. S3 and [28]. The other two mutants, Z(F193E) and Z(K194A), are slightly to moderately impaired in activating viral replication and late gene expression (Fig. 1, [28] and unpublished data). 48 h after transfection of BZKO cells, we compared the level of *balf2* expression among the mutants using Northern blot analysis. All five mutants activated the *balf2* message to a level equivalent to wt ZEBRA (Fig. 3A). As a positive control for migration of the *balf2* transcript we used RNA from HH514-16 cells induced into the lytic cycle with sodium butyrate.

Using quantitative RT-PCR we assessed the level of transcripts encoding the heterotrimeric helicase-primase complex in cells expressing five RD mutants: the regulatory mutants, Z(S173A) and Z(S167A/S173A) and the three basic domain mutants, Z(Y180E), Z(R187K) and Z(K188A). We employed two different methods to prepare cDNA from purified RNA samples. In the experiment illustrated in Fig. 3B and 3C, we synthesized cDNA using gene specific primers that were complementary to viral helicase (BBLF4) or viral primase (BSLF1). In Fig. 3D, 3E and 3F, we used a mixture of random hexamers and poly-dT to synthesize cDNA. It is important to note that each of the DNA fragments amplified by RT PCR acquired the same melting point and electrophoretic mobility on agarose gels as DNA fragments amplified by PCR from an expression vector containing a cloned version of the corresponding gene (data not shown). To confirm that the purified RNA samples were not contaminated with genomic DNA we omitted the reverse transcriptase enzyme from the reaction mixture. As a result no DNA amplification was detected (Fig. 3B).

Regardless of the method used for cDNA preparation, we found that the levels of mRNAs for viral helicase, primase and primase-associated factor (BBLF2/3) in cells expressing wt ZEBRA were several fold higher than in cells transfected with empty vector. All four RD mutants were competent to activate expression of the viral helicase and primase to levels comparable or higher than those activated by wt ZEBRA. The mutants, particularly the basic domain mutants, activated twice as much helicase and primase transcripts as the wild type protein. For example, Z(K188A) activated between 2.3 to 2.6-fold more *bblf4* mRNA than wt ZEBRA (Fig. 3B and 3D).

### Expression of BBLF2/3 is insufficient to suppress the phenotype of the ZEBRA RD mutants

**Figure 4:**
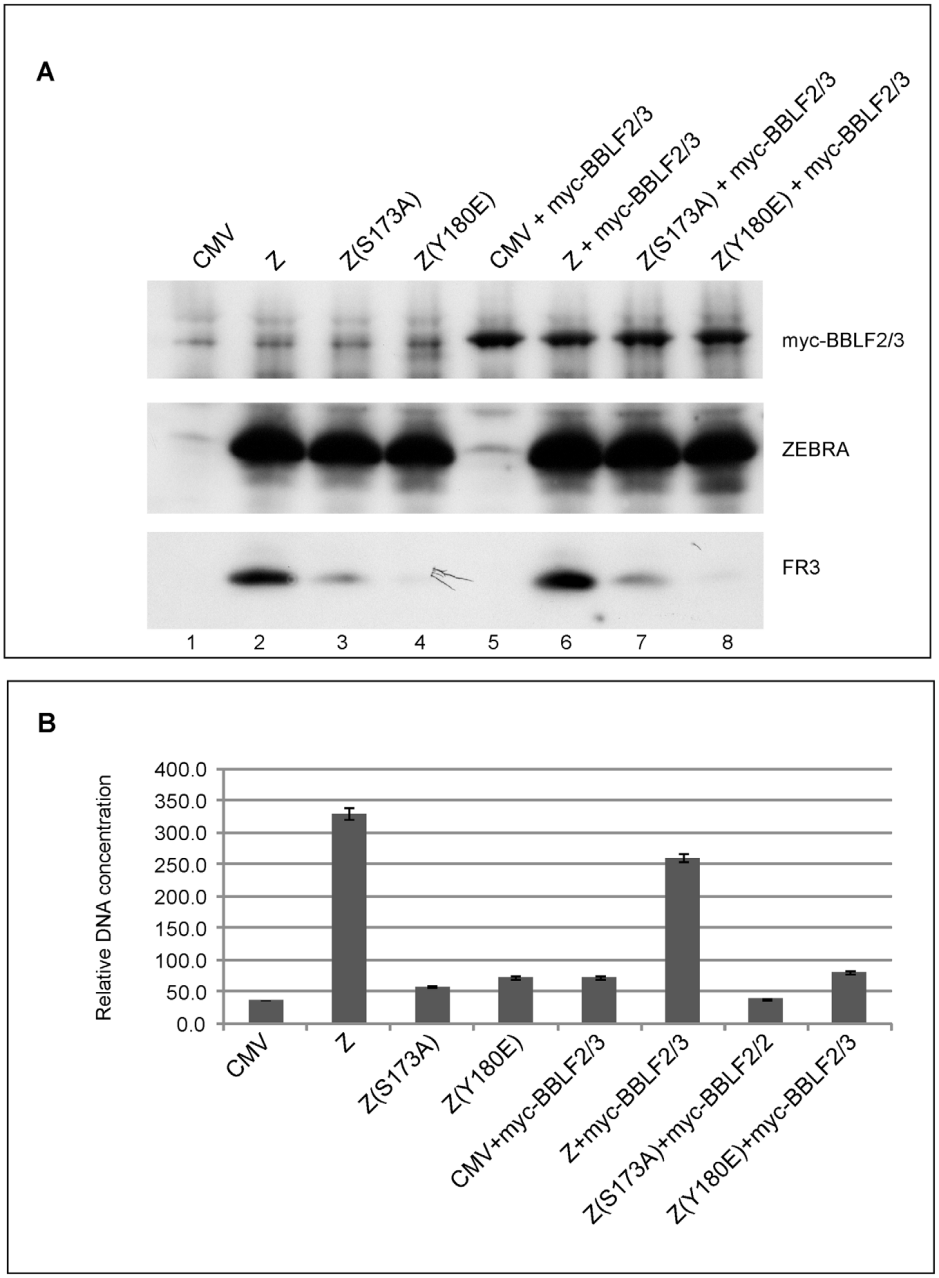
Over-expression of BBLF2/3 fails to complement the defect in ZEBRA mutants impaired in lytic viral replication and late gene expression. A) Western blot analysis of BZKO cells expressing the indicated proteins. The membrane was incubated with antibodies against BFRF3, ZEBRA and the myc tag. B) Real time PCR to detect the extent of viral genome amplification in BZKO cells transfected with the indicated ZEBRA replication defective mutants in the absence or presence of BBLF2/3. The primers were specific for the EBV *brlf1* promoter. The data shown represent the average of three different experiments.

The primase-associated-factor (BBLF2/3) was the only gene that exhibited lower transcript levels in cells expressing RD mutants compared to those expressing wt ZEBRA (Fig. 3F). However, the level of *bblf2/3* mRNA was still 5–9-fold above background. To determine whether ectopic expression of BBLF2/3 could rescue the defect in these mutants, we co-expressed BBLF2/3 with two ZEBRA RD mutants, Z(S173A) and Z(Y180E), in BZKO cells. Forty-eight hours after transfection, cells were harvested and analyzed for late gene expression and viral replication. We found that over-expression of BBLF2/3 had no effect on the level of the late protein, FR3, induced by wt ZEBRA, Z(S173A) or Z(Y180E) (Fig. 4A). Similarly, using quantitative PCR to determine the extent of viral genome amplification, we found the same levels of viral genome in cells expressing Z(S173A) or Z(Y180E) in the absence or presence of BBLF2/3. The level of viral DNA present in cells transfected with the mutants was approximately equal to that in control cells transfected with empty vector (Fig. 4B). These experiments showed that impairment of ZEBRA RD mutants to induce late gene expression and viral replication was not the result of the slightly reduced levels of the *bblf2/3* transcript detected following expression of this class of ZEBRA mutants. Moreover, over-expression of BBLF2/3 protein could not rescue the late mutants.

### ZEBRA RD mutants display weak DNA binding activity *in vitro*

**Figure 5:**
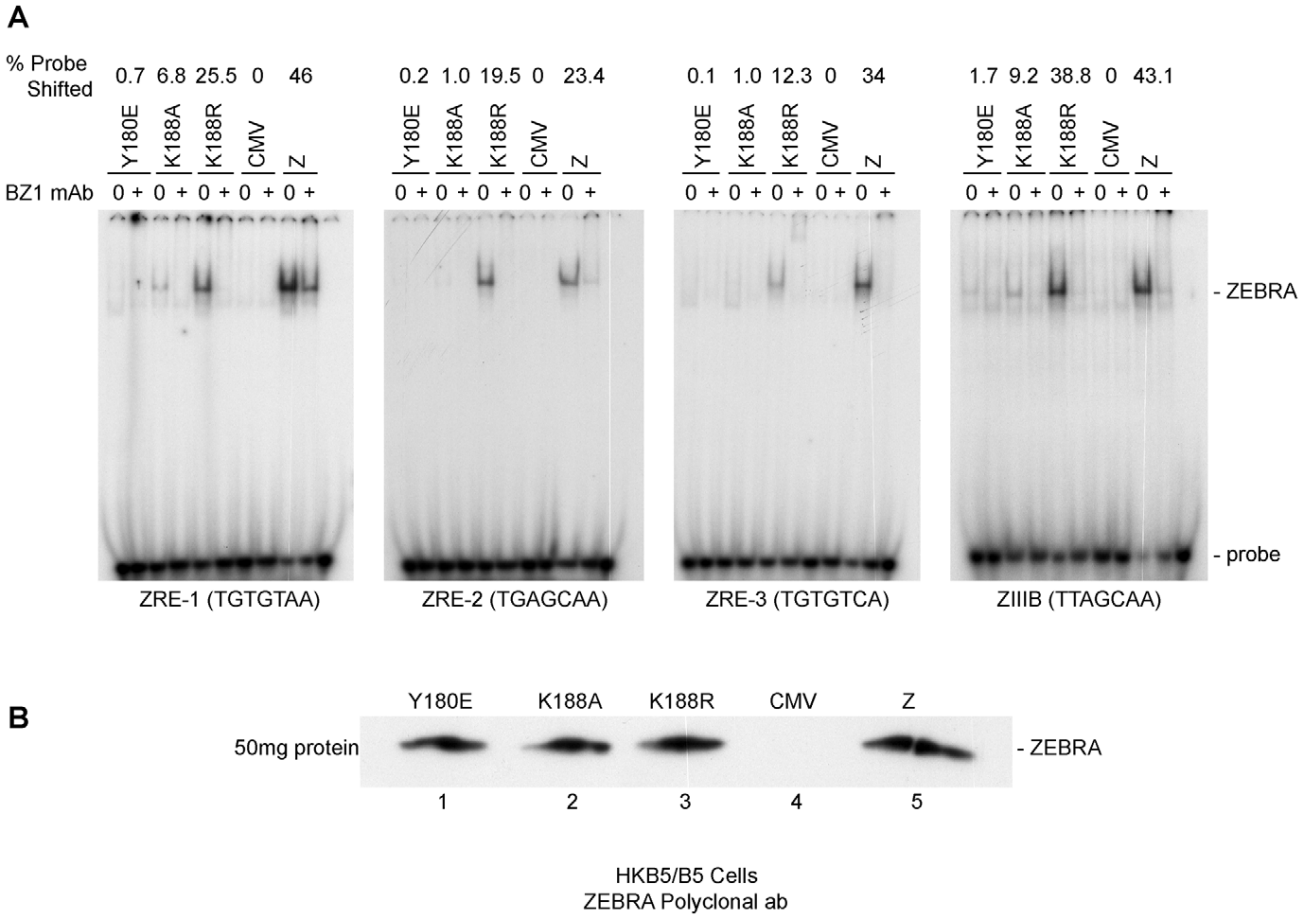
ZEBRA mutants that fail to activate viral replication are defective at binding DNA *in vitro*. A) Electrophoretic mobility shift assay (EMSA) comparing the DNA binding activity of Z(Y180E), Z(K188A), Z(K188R) and wt ZEBRA using four ZEBRA response elements. ZREs 1-3 are present in the origin of lytic replication (oriLyt), while ZIIIB is present in the *bzlf1* promoter. The latter site has the highest affinity to ZEBRA among other known ZREs and was used as a positive control for binding. Probes were shifted using HKB5/B5 cell extracts expressing different ZEBRA proteins. B) Western blot analysis of the levels of wild-type ZEBRA or mutant ZEBRA present in cell extracts used for EMSA. The immunoblot was probed with a polyclonal antibody against ZEBRA.

Previously, we showed that reduction in the DNA binding activity of ZEBRA, due to alanine substitution of the phosphorylation site S173, correlated with a defect in the capacity of ZEBRA to induce viral replication. The same impairment of binding was detected between Z(S173A) and the Rta promoter, but Z(S173A) was competent to activate expression of Rta to the same extent as wt ZEBRA [25]. This finding provoked the hypothesis that the DNA binding affinity of ZEBRA was of relatively greater importance for activation of viral replication than for activation of transcription. To further investigate this correlation we used an electrophoretic mobility shift assay (EMSA) to assess the DNA binding activity of ZEBRA RD mutants located in the basic domain of the protein. Fig. 5 compares the DNA binding activity of Z(Y180E) and K(188A) with that of wt ZEBRA and with Z(K188R), a mutant with a conservative change that manifests a wild phenotype. An EMSA assay was performed using cell extracts obtained from EBV negative HKB5/B5 cells transfected with the indicated expression vectors. Four ZEBRA response elements, ZIIIB and ZREs 1 to 3, were used as probes. ZIIIB represents the highest affinity binding site for ZEBRA; it mediates auto-stimulation of the ZEBRA promoter [37], [38]. ZREs 1–3 represent a cluster of sites present in the upstream essential region of oriLyt. Both Z(Y180E) and Z(K188A) were markedly impaired in binding to each of the four probes relative to wt ZEBRA. The efficiency of binding was calculated as the percentage of probe shifted by each mutant protein. Z(Y180E) shifted between 0.1% and 0.7% depending on the probe used in the shift assay; Z(K188A), 1% to 9.2%, and wt ZEBRA, 23.4% to 46% (Fig. 5A). The ZEBRA mutant, Z(K188R), which is fully competent to activate the lytic cycle [28], shifted the same set of ZEBRA specific DNA probes to percentages that were markedly higher than those observed with the ZEBRA RD mutants, namely 12.3% and 38.8% of the total probe (Fig. 5A). These *in vitro* DNA binding studies clearly indicated that Z(Y180E) and Z(K188A) are both significantly impaired in their capacity to bind to ZEBRA response elements present in regulatory sites for transcription or replication. The differences in DNA binding between wt ZEBRA and the mutants were not due to variable protein levels. Western blot analysis with an antibody against ZEBRA demonstrated that all EMSA extracts contained similar levels of ZEBRA protein (Fig. 5B).

### ZEBRA RD mutants interact weakly with oriLyt *in vivo*

**Figure 6 ppat-1001054-g006:**
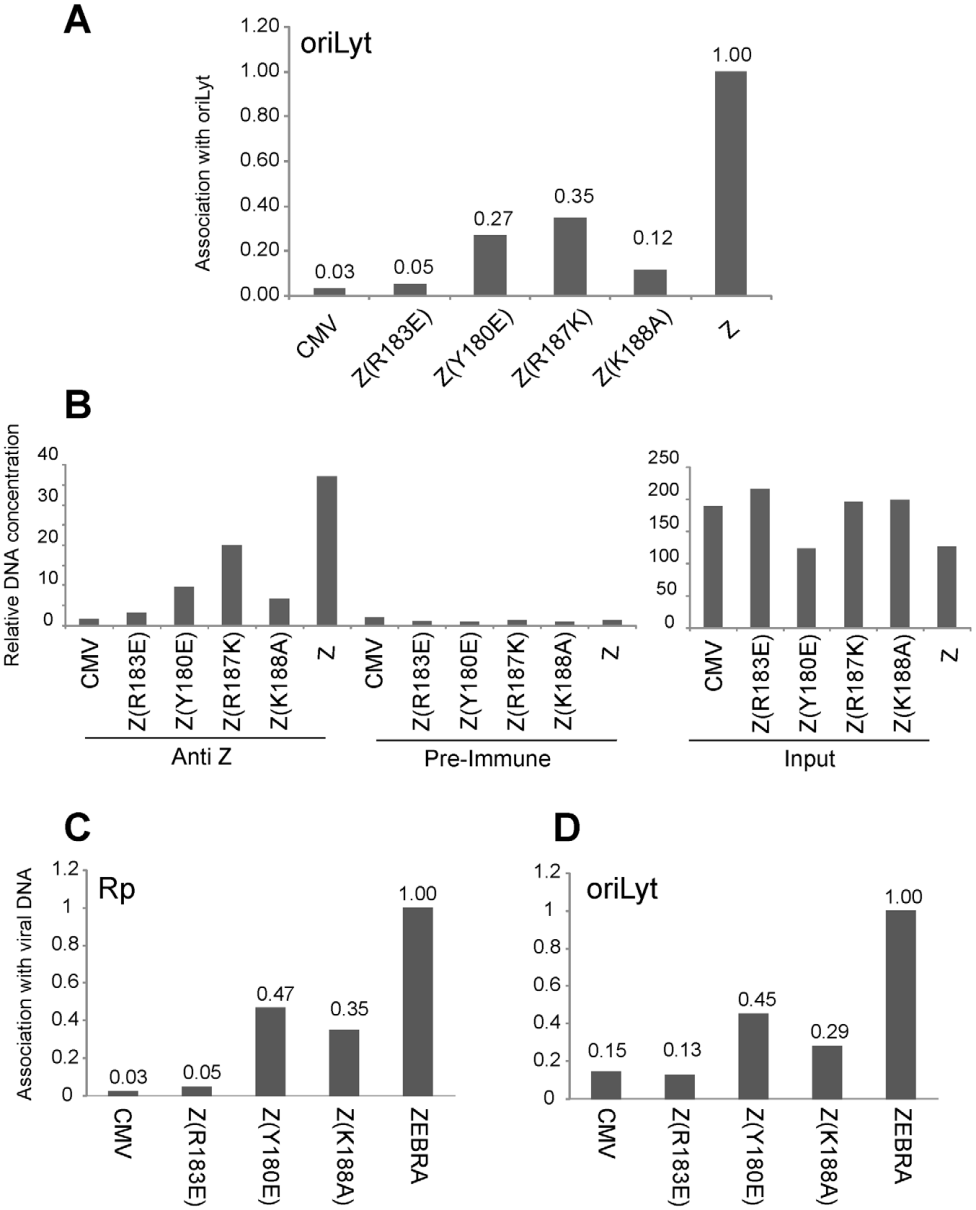
Replication defective ZEBRA proteins with single point mutations in the basic domain associate weakly with oriLyt and Rp *in vivo*. Chromatin immunoprecipitation of ZEBRA protein and its associated DNA. BZKO cells were transfected with expression vectors encoding wt ZEBRA, a non-DNA binding mutant Z(R183E), and three replication defective ZEBRA mutants [Z(Y180E), Z(R187K) and Z(K188A)]. PAA was added to block viral DNA replication. Cells were harvested 48 h after transfection. (A) The amount of precipitated oriLyt was measured by real time PCR using the standard curve method. The bar graph represents the amount of oriLyt precipitated by a polyclonal ZEBRA antibody divided by the amount of oriLyt detected in the corresponding input sample (panel B). The ChIP/Input value of each sample was normalized to that of wild type ZEBRA (Z). B) Relative amount of oriLyt DNA either present in input samples or immunoprecipitated by pre-immune or anti ZEBRA antibody. (C) and (D) Comparison of the association of ZEBRA replication defective mutants with Rp and oriLyt. Chromatin immunoprecipitation of ZEBRA protein from BZKO cells expressing wt ZEBRA, Z(R183E), Z(Y180E) or Z(K188A) and treated with PAA. Quantitative PCR was performed using primers specific to Rp (C) and to oriLyt (D). The amount of ZEBRA-associated Rp or oriLyt was corrected for the total level of Rp or oriLyt present in the corresponding input sample. Panels A and B and panels C and D represent data from two separate ChIP experiments.

To analyze the ability of the ZEBRA RD mutants to associate with the viral origin of lytic replication (oriLyt) *in vivo*, we employed chromatin immunoprecipitation (ChIP). To study the associations of the three basic domain ZEBRA RD mutants with oriLyt we transfected BZKO cells with expression vectors encoding each of the ZEBRA RD mutants, wt ZEBRA and a non-DNA binding form of ZEBRA, Z(R183E), which does not activate transcription or replication. In this experiment, the wild type protein was the only form of ZEBRA that was capable of inducing viral replication. To compare the amount of oriLyt immunoprecipitated by each ZEBRA protein we maintained equivalent levels of viral DNA by blocking viral replication with phosphonoacetic acid (PAA). We found that all ZEBRA RD mutants were more efficient than the non-DNA binding mutant Z(R183E), but less competent than wt ZEBRA in precipitating the upstream region of oriLyt. 3.7-fold less oriLyt was immunoprecipitated from cells expressing Z(Y180E) compared to those expressing wt ZEBRA (Fig. 6A). Similarly, Z(R187K) and Z(K188A) pulled down 2.9 and 8.3-fold less DNA than wt ZEBRA. The extent of association of each mutant with oriLyt was corrected for the total amount of oriLyt detected in the corresponding input sample. Fig. 6B shows that the level of input oriLyt was approximately the same in cells transfected with wild type and all three mutants. These results suggest that amino acid changes introduced in the three ZEBRA RD mutants did not completely abolish interaction of ZEBRA with oriLyt as was observed with the non DNA binding mutation R183E. Nonetheless, the ability of the RD mutants to bind to oriLyt in cells was 3- to 8-fold impaired compared with wt ZEBRA.

### Replication defective mutants are similarly impaired in interacting with Rp and with oriLyt

The Rta promoter (Rp) is a direct target for activation by ZEBRA. In Fig. 1, 2, S4 and S5, we showed that all ZEBRA RD mutants were fully competent to induce wild type levels of *brlf1* (Rta) mRNA and protein. However, EMSA experiments showed that the ZEBRA RD mutants were similarly defective in binding to ZEBRA response elements regardless of their presence in transcription or replication regulatory regions (Fig. 5 and [25]). To investigate whether the ZEBRA RD mutants are impaired in their capacity to associate with Rp, in a separate experiment we carried out ChIP experiments to compare directly the capacity of two RD mutants to precipitate oriLyt and Rp DNA relative to wt ZEBRA. We found that Z(Y180E) and Z(K188A) were two-to three-fold defective in interacting both with Rp and with oriLyt when compared to the wild type protein (Fig. 6C and D). However, these RD mutants displayed higher efficiency to interact with oriLyt and Rp than the non-DNA binding ZEBRA mutant Z(R183E). The Z(R183E) mutant pulled down amounts of oriLyt and Rp that were equivalent to those detected in ChIP experiments performed with cells transfected with empty vector or precipitated with pre-immune serum (Fig. 6). The finding that RD mutants are equally impaired in binding to Rp and oriLyt suggests that activation of *brlf1* transcription is more tolerant of weaker interaction between ZEBRA and its response elements than is stimulation of replication.

### Assessing the association of ZEBRA with oriLyt and Rp using an *in vivo* biotinylated DNA affinity assay (iBDAA)

**Figure 7:**
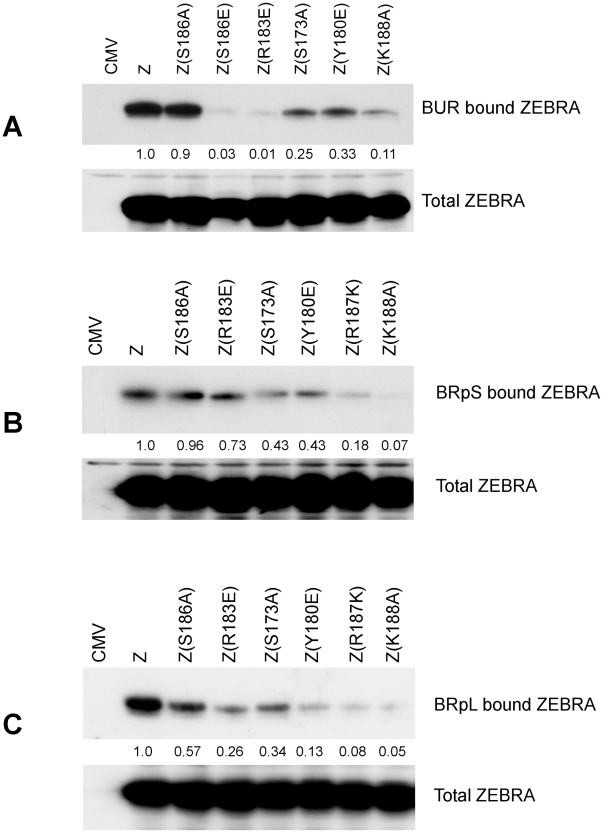
ZEBRA RD mutants share a common defect in interacting *in vivo* with Biotinylated probes containing regions of oriLyt and *brlf1* promoter (Rp). The capacity of ZEBRA mutants to bind to the upstream region of oriLyt [BUR] (panel A), a short region of Rp [BRpS] (panel B) and full length Rp [BRpL] (panel C) was compared. BZKO cells were transfected with expression vectors for the indicated forms of ZEBRA together with Biotinylated oligomers containing one of these three regulatory regions. The Biotinylated probes were purified from cell lysates using avidin beads. The amount of ZEBRA bound to each probe and the total amount of ZEBRA present in each lysate were assessed by western blot analysis. Relative binding was calculated by comparing the amount of ZEBRA pulled down by the probe corrected for the total amount of ZEBRA detected in input samples.

The ChIP assay measures the amount of DNA associated with ZEBRA, but does not measure how much ZEBRA interacts with DNA. Therefore, we employed a different approach to assay for the capacity of ZEBRA to bind DNA in cells (Fig. 7). The assay relied on co-transfecting vectors encoding wild type ZEBRA or ZEBRA mutants together with biotin-conjugated probes. The BUR probe is 167 bp long and encompasses the four ZREs in the upstream region of oriLyt that are crucial for lytic replication. BRpS and BRpL represent short (156 bp) and long (277 bp) segments of Rp. BRpS contains the ZIIIA site, while the BRpL has all three identified ZREs present in Rp. After 48 h, BZKO cells were harvested and biotinylated probes were captured using avidin coated beads. The level of ZEBRA protein bound to each probe was determined by western blot. The relative binding of ZEBRA to each probe was corrected for the total amount of ZEBRA protein present in each sample. In cells transfected with ZEBRA RD mutants, all three biotinylated probes pulled down less ZEBRA protein compared to cells transfected with wt ZEBRA. The defect in binding relative to wt ZEBRA averaged between 75% to 89% for the oriLyt probe (Fig. 7A); 57% to 93% for the short Rp probe (Fig. 7B), and 66% to 95% for long Rp probe (Fig. 7C). Our results with the transfected biotinylated probe assay confirm the EMSA and ChIP experiments. These three different assays show that replication defective mutants of ZEBRA are markedly impaired in binding to DNA. This defect in DNA binding can be seen with probes for oriLyt and Rp.

### Expression of the six EBV replication proteins partially restored the capacity of RD mutants to activate viral replication and late gene expression

**Figure 8:**
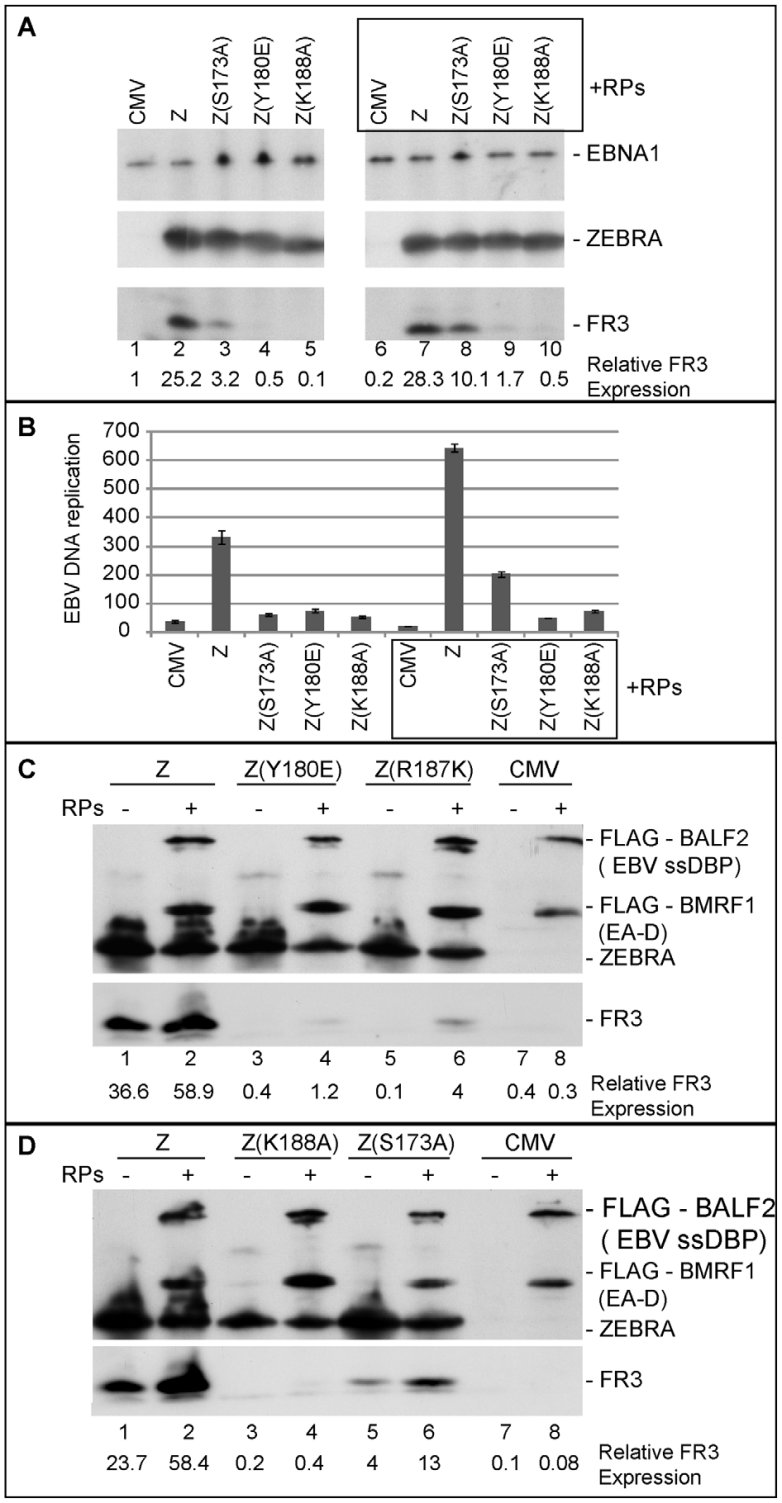
Over-expression of EBV replication proteins partially suppresses the phenotype of Z(S173A) and Z(R187K). A) Immunoblot demonstrating the effect of EBV replication proteins on late gene expression in BZKO cells expressing Z(S173A), Z(Y180E), and Z(K188A). The membrane was blotted with antibodies against the indicated viral proteins FR3, ZEBRA and EBNA1. B) Replication assay to measure the extent of viral genome amplification induced by wt ZEBRA and the indicated ZEBRA mutants in the absence or presence of a mixture of expression vectors encoding the six EBV replication proteins. C and D) Immunoblots of BZKO cell extracts transfected with empty vector or expression vectors encoding wt ZEBRA or the indicated ZEBRA RD mutants with and without the six core EBV replication proteins. The membrane was blotted with antibodies specific to FLAG tag, ZEBRA and FR3.

Our findings suggest that weak association of ZEBRA with oriLyt has significant ramifications for subsequent events that lead to lytic viral DNA replication. These events might involve a specific protein-protein interaction between ZEBRA and one or more of the replication proteins. In an attempt to restore this interaction we over-expressed the six components of the EBV replication machinery together with each of the ZEBRA RD mutants in BZKO cells. Over-expression of replication proteins partially rescued late gene expression by all four ZEBRA RD mutants. The extent of rescue ranged between 3- to 4-fold regardless of the level of late gene expression induced by each mutant in the absence of replication proteins (Fig. 8A, C and D). For example, in case of Z(S173A), expression of replication proteins reproducibly increased FR3 expression by 3.2-fold reaching 55% that of wt ZEBRA alone. This effect on late gene expression was not an anomalous feature of these mutants; a similar increase was detected with the wild type ZEBRA protein and ranged between 1.6- and 2.5-fold (Fig. 8C and D). While expression of the late FR3 protein can be used as an indirect marker for viral replication, we also examined the effect of over-expressing replication proteins on the capacity of wt ZEBRA and ZEBRA RD mutants to induce viral genome amplification. Expression of high levels of replication proteins reproducibly augmented the capacity of wt ZEBRA and Z(S173A) to stimulate EBV lytic replication by 1.9- and 3.4-fold respectively. In this experiment no similar effect of the complete mixture of replication proteins on DNA amplification was observed with the other ZEBRA RD mutants (Fig. 8B). However, subsequent experiments defined a subset of replication proteins that was capable of rescuing replication by all the RD mutants (Fig. S3).

### Expression of EBV replication proteins increased ZEBRA association with oriLyt *in vivo*

**Figure 9:**
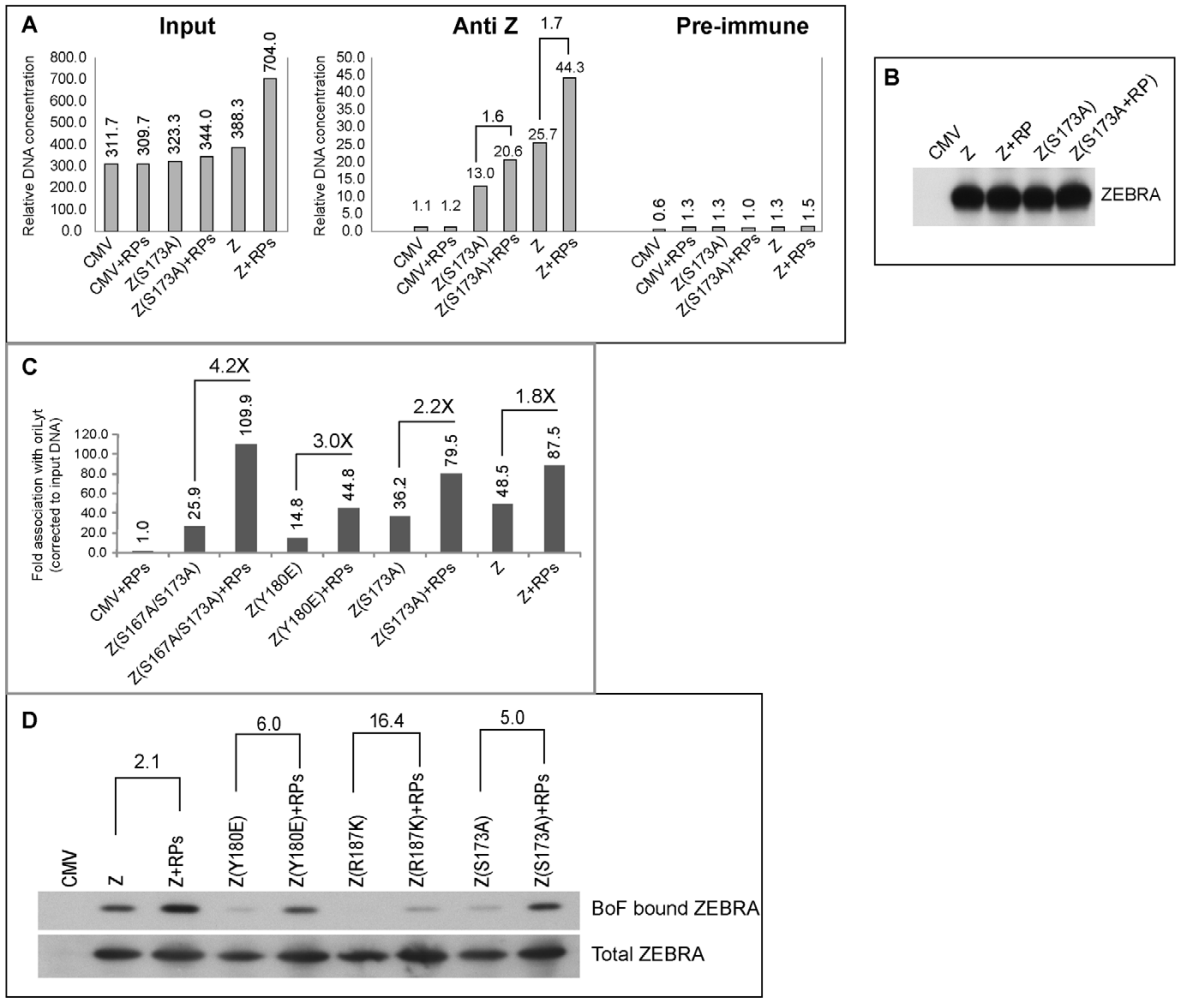
EBV replication proteins enhance association of ZEBRA with oriLyt. A) ChIP comparing the level of ZEBRA-bound oriLyt in the absence and presence of replication proteins. Real time PCR was employed to detect the level of co-immunoprecipitated oriLyt using primers complementary to the upstream region. B) Immunoprecipitation of ZEBRA proteins from BZKO cell extracts using the same conditions followed in ChIP. C) A biological replicate experiment using chromatin immunoprecipitation of wt ZEBRA and the indicated ZEBRA mutants to study the effect of expressing a mixture of EBV replication proteins on the interaction between ZEBRA and oriLyt. Fold association with EBV oriLyt DNA was corrected for the amount of input oriLyt. D) Western blot analyses showing the amount of ZEBRA proteins either pulled down by biotinylated full length oriLyt (BoF) (upper panel) or present in input samples (lower panel), in the absence and presence of replication proteins.

In experiments illustrated in Figs. 5 to [Fig ppat-1001054-g006] 7 and previously published [25] we found a direct correlation between strong association of ZEBRA with oriLyt and viral replication. To explore the possibility that replication proteins enhance interaction of ZEBRA with oriLyt, thereby partially restoring EBV lytic replication, we carried out ChIP experiments combined with quantitative PCR. In Fig. 9A, BZKO cells were transfected with empty vector (CMV), Z(S173A) or wt ZEBRA in the presence and absence of the six EBV replication proteins. In ChIP experiments, we found that BZKO cells transfected with Z(S173A) or wt ZEBRA yielded more oriLyt when replication proteins were co-expressed, 1.58-fold and 1.72-fold, respectively (Fig. 9A). This increase was independent of the level of ZEBRA expressed or immunoprecipitated. Western blot analysis showed that similar levels of ZEBRA protein were present in each immune-precipitate (Fig. 9B). Expression of the six replication proteins had no effect on the amount of oriLyt immunoprecipitated from cells transfected with empty vector.

A biological replicate experiment was performed and included two additional ZEBRA RD mutants, Z(Y180E) and Z(S167A/S173A) [25]. Wild type and mutant ZEBRA were expressed in BZKO cells minus and plus replication proteins. Co-expression of replication proteins enhanced the ability of wild type and mutant forms of ZEBRA protein to associate with oriLyt *in vivo*. A 1.8-fold increase in association with oriLyt was detected with wt ZEBRA; 2.2-fold with Z(S173A); 3-fold with Z(Y180E), and 4.24-fold with Z(S167A/S173A) (Fig. 9C).

A compilation of several chromatin immunoprecipitation experiments showed that replication defective ZEBRA mutants weakly associated with oriLyt (Fig. S2A). Z(S173A) was the least defective while Z(K188A) was the most impaired. For wild type ZEBRA and three of the mutants, Z(S173A), Z(Y180E) and Z(S167A/S173A), we demonstrated an increase in their association with oriLyt as a result of overexpressing the EBV replication proteins. The effect of replication proteins on association of ZEBRA with oriLyt was greatest with the mutant Z(S167A/S173A), 6.87-fold. Z(Y180E) precipitated 3 times more oriLyt in the presence replication proteins; Z(S173A), 1.8-fold, and wild type ZEBRA 1.6-fold (Fig. S2A). The two ZEBRA RD mutants which were most defective in binding to oriLyt, namely Z(R187K) and Z(K188A) were the least affected by replication proteins.

To investigate further the effect of replication proteins on interaction of ZEBRA with oriLyt we transfected BZKO cells with Biotin-conjugated oriLyt Full length (BOF) and expression vectors encoding wild type and the RD ZEBRA mutants with and without replication proteins. Cells were harvested 48 h after transfection; ZEBRA bound to oriLyt was purified using avidin coated beads. Both input and BoF-captured ZEBRA proteins were analyzed by Western blot. The effect of replication proteins on binding of ZEBRA to oriLyt was calculated after correcting for the amount of ZEBRA present in the corresponding input samples. We found that co-expression of the six core components of the replication machinery enhanced binding of wt ZEBRA, Z(Y180E), Z(R187K) and Z(S173A) to oriLyt by 2.1-, 6.0-, 16.4- and 5.0-fold, respectively. In summary the ChIP and iBDAA experiments demonstrate that the core components of the EBV replication machinery augment the interaction between ZEBRA and oriLyt.

### Replication proteins enhanced interaction of ZEBRA with EBV early lytic cycle promoters

To determine whether the effects of replication proteins were specific for ZEBRA's association with oriLyt, we examined the effect of replication proteins on association of ZEBRA with other lytic viral regulatory sites by studying interaction of ZEBRA and RD mutants with Rp and two other ZEBRA responsive promoters, BZLF1p (Zp) and BMRF1p (EAp). Zp is auto-stimulated by ZEBRA while BMRF1p is activated by synergy between ZEBRA and Rta. Over-expression of the six EBV replication proteins increased the relative amount of Rp, Zp and BMRF1p DNA precipitated by wt ZEBRA, Z(S167A/S173A) and Z(S173A) (Fig. S2B and Supplementary Table S1). The effect of replication proteins on the amount of viral DNA pulled down by Z(Y180E) was more pronounced on Rp (2.2-fold). The amount of Z(Y180E) bound to Zp and BMRF1p was minimally enhanced by replication factors, 1.3-fold and 1.25-fold, respectively. No difference was detected by ChIP for the effect of replication proteins on the relative binding capacity of Z(R187K) and Z(K188A) to Rp. This could be attributed to the marked defect in the DNA binding capacity of these two mutants or limitations in the ChIP technique to detect small changes in association with a particular site. Our results show that replication proteins enhance the interaction of ZEBRA and the phosphorylation site mutants with oriLyt, and with at least three transcription regulatory sites, Rp, Zp and EAp.

### Three replication proteins are sufficient to rescue the functional defect in ZEBRA RD mutants

To delineate the contribution of each replication protein in restoring lytic viral DNA synthesis, Z(S173A) was co-expressed with different mixtures of replication proteins. In each mixture one of the six components was omitted. After 48 h, DNA was purified from BZKO cells and analyzed for its viral DNA content using quantitative PCR (Fig. S1). Elimination of individual components of the mixture of replication proteins led to several distinct outcomes. Exclusion of BBLF2/3 had no significant effect. Omission of BALF2 and BBLF4 reduced the efficacy of the replication proteins complex to rescue replication by Z(S173A). Eliminating BSLF1 or BMRF1 from the mixture of replication proteins abolished its activity. In contrast, omitting the expression vector of BALF5 augmented the capacity of the other five replication proteins to restore viral replication by Z(S173A). These results suggest that over-expression of different mixtures of replication proteins can stimulate, inhibit or have no effect on viral replication.

**Figure 10:**
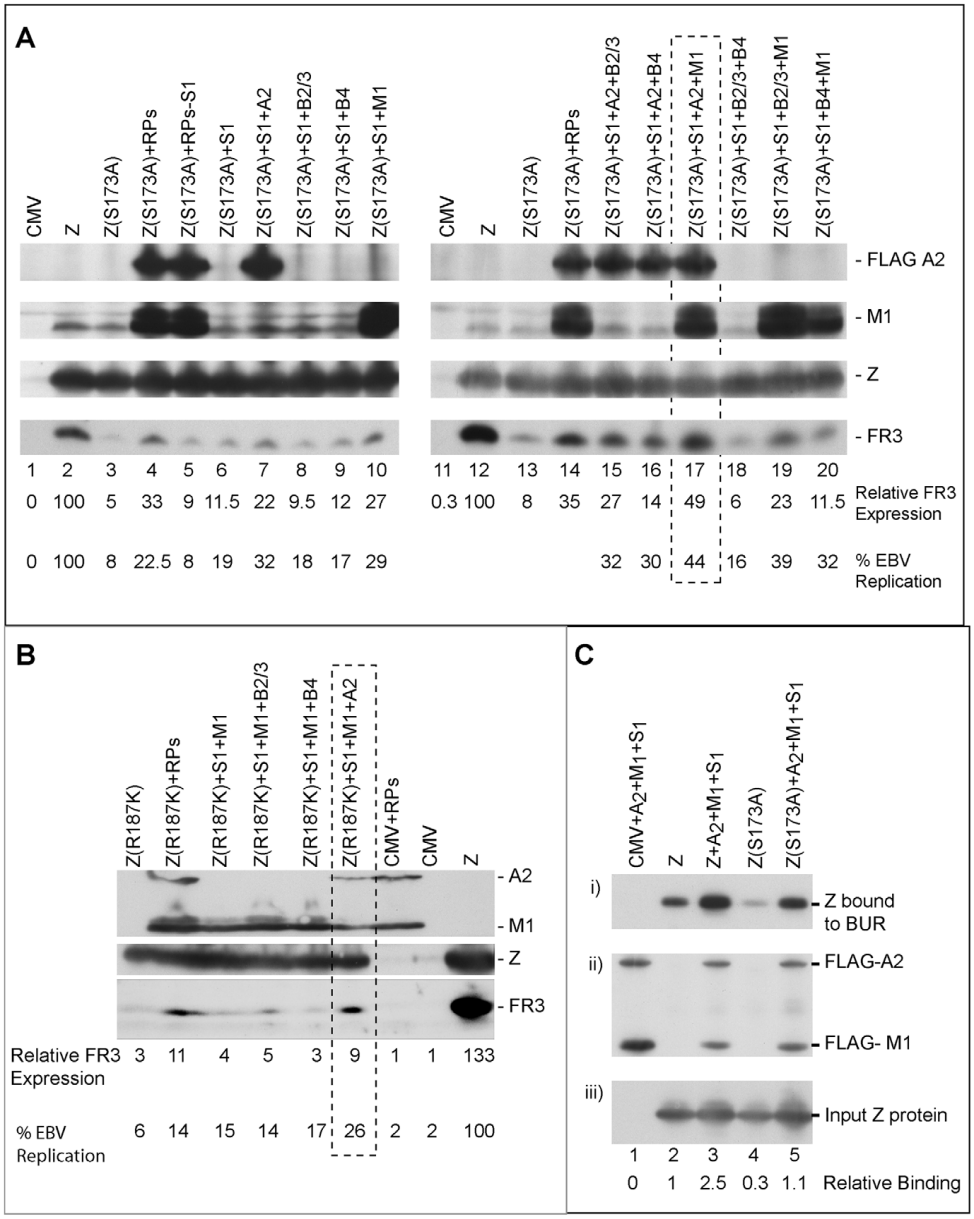
Three replication proteins are sufficient to rescue the late gene expression defect in the ZEBRA RD mutants. (A) Immunoblot of BZKO cell extracts expressing empty vector (CMV), wt ZEBRA (Z) or Z(S173A) in the absence and presence of various combinations of replication proteins. The membrane was probed for FLAG-BALF2 (ssDNA-binding protein), M1 (DNA processivity factor), ZEBRA (Z) and FR3 (the viral capsid antigen BFRF3). Relative late gene expression and viral genome amplification were calculated by setting the levels induced by wt ZEBRA as 100%. (B) The effect of replication proteins on late gene expression and viral replication activated by Z(R187K). The membrane was blotted for BALF2 (A2) and BMRF1 (M1) simultaneously using a FLAG antibody. Expression of ZEBRA (Z) and FR3 was determined using specific antibodies. (C) Interaction of ZEBRA with the upstream region of oriLyt was markedly enhanced when (S1) the viral primase (BSLF1), (A2) the ssDNA-binding protein (BALF2) and (M1) the polymerase associated factor (BMRF1) were over-expressed. Panel (i) depicts the amount of ZEBRA captured by biotinylated upstream region of oriLyt (BUR). Panel (ii) depicts ssDNA-binding protein and polymerase associated factor while panel (iii) portrays the total amount of ZEBRA present in input samples. The ssDNA-binding protein and the polymerase associated factor were both detected using a FLAG monoclonal antibody. Relative binding of ZEBRA to BUR was corrected for the corresponding amount of input protein. RPs, replication proteins; B2/3, the primase associated factor (BBLF2/3), and B4, the helicase (BBLF4).

To select the minimum subset of replication proteins sufficient to suppress the phenotype of these RD ZEBRA mutants, we examined the effect of expressing the primase individually or together with various combinations of replication proteins excluding the polymerase (BALF5) that had been shown to be inhibitory (Fig. S1). After 48 h, transfected BZKO cells were analyzed by Western blot for the level of the FR3 protein as a marker for late gene expression. While co-expression of all six replication proteins with Z(S173A) induced late gene expression to 33.4 and 35.4% that of wt ZEBRA (Fig. 10A compare lane 3 to 4 and 13 to 14), addition of the primase alone had no significant effect on the level of the FR3 protein as compared to cells transfected with the S173A mutant in absence of RP. However, combining the primase with either the viral single-stranded DNA binding protein (BALF2) or the viral DNA polymerase processivity factor (BMRF1) enhanced late gene expression to 21.8 and 27.2% of wild type, respectively. A mixture containing all three proteins, the primase, the ssDNA-binding protein and the DNA polymerase processivity factor, restored late gene expression to 49.1%, a level higher than that induced by all six replication proteins (Fig. 10A lane 17). Addition of the viral helicase and/or the primase associated factor was either inhibitory or had no effect on the level of FR3.

To assess the effect of the different combinations of replication proteins on viral replication, we purified DNA from the same group of cells and analyzed it using quantitative PCR. The findings obtained by qPCR were similar to those seen by analyzing late gene expression. A mixture of the primase, the single-stranded DNA binding protein and the DNA polymerase processivity factor suppressed the defect in Z(S173A) and restored replication to approximately 44% that of the level activated by the wild type protein (Fig. 10A).

To determine if the same tripartite mixture of replication proteins could complement the defect in viral genome amplification observed in ZEBRA mutants in the DNA recognition domain, we repeated the same experiment using Z(R187K). Addition of all replication proteins induced viral replication 2.2-fold above that induced by Z(R187K) alone. Transfection of the primase and the DNA polymerase processivity factor together with Z(R187K) had no effect on late gene expression or viral DNA synthesis (Fig. 10B). However, addition of the single-stranded DNA binding protein to this mixture resulted in the highest impact on viral genome amplification, a 4.2-fold increase compared to replication induced by Z(R187K) alone (Fig. 10B). Similar results were observed for the effect of these three replication proteins on late gene expression (Fig. 10B).

The capacity of BALF2, BMRF1 and BSLF1 to rescue viral genome amplification by all five identified ZEBRA RD mutants was examined. BZKO cells were transfected with expression vectors encoding Z(S167A/S173A), Z(S173A), Z(Y180E), Z(R187K), Z(K188A) and wild type ZEBRA in the absence and presence of plasmids encoding the tripartite mixture of replication proteins. Cells were harvested at 48 h and 72 h and DNA was purified. The amount of EBV lytic replication induced by each condition was assessed by qPCR. At both time points, over-expression of the three components of the replication machinery enhanced activation of EBV lytic DNA replication by all five replication defective mutants as well as wt ZEBRA (Fig. S3).

Our findings stress the importance of the primase, the DNA polymerase processivity factor and the single-stranded DNA binding protein on suppressing the effect of ZEBRA mutations that render the protein incompetent to activate lytic DNA replication.

### The tripartite replication mixture enhanced ZEBRA interaction with the upstream region of oriLyt

The upstream region of oriLyt encompasses four ZEBRA binding sites that are essential for oriLyt replication. Therefore it was important to assess directly the effect of the three replication proteins that rescued the function of the RD mutants on the capacity of ZEBRA to interact with the upstream region of oriLyt. In an iBDA assay, we transfected BZKO cells with a biotinylated upstream region of oriLyt (BUR) together with expression vectors for wt ZEBRA or Z(S173A) in the absence and presence of the tripartite replication mixture. BUR-bound ZEBRA was captured on avidin coated beads and the amount of ZEBRA bound was analyzed by western blot. We found that over-expression of the primase, the ssDNA-binding protein and the polymerase associated factor resulted in a 2.5- to 3.7-fold increase in the amount of ZEBRA that interacted with BUR (Fig. 10C). This finding supports a role for the tripartite mixture of replication proteins in lytic origin recognition by ZEBRA.

### Expression of the tripartite mixture of BALF2, BMRF1 and BSLF1 co-activate transcription of *brlf1*, the gene encoding Rta, by ZEBRA mutants

The results presented in supplemental Fig. S2B show that over-expression of replication proteins enhanced the capacity of wt ZEBRA, the phosphorylation site mutants and Z(Y180E) to interact with Rp, the BRLF1 promoter. The functional significance of expressing this subset of replication proteins on transcriptional activation of *brlf1* by wt ZEBRA or mutant ZEBRA was studied in BZKO cells. To maintain an equal number of viral genome templates in each group, viral replication was blocked by phosphonoacetic acid (PAA) and the cells were harvested after 24 hours. Total RNA was purified and the level of *brlf1* transcript was assessed using quantitative RT-PCR. Fig. S4A represents the average of two biological replicate experiments in which each value is the mean of three distinct RT-PCR reactions. As previously demonstrated in Fig. 2, expression of ZEBRA replication defective mutants induced the synthesis of up to 2.4-fold more *brlf1* mRNA than did wt ZEBRA. Over-expression of the tripartite mixture of replication proteins co-stimulated synthesis of the *brlf1* transcript to various levels depending on the form of the ZEBRA protein being expressed. Replication proteins had a modest effect on the capacity of wt ZEBRA, Z(S173A) and Z(S167A/S173A) to activate transcription of *brlf1* (1.3 to 1.5- fold). A significant 2.4-fold to 3.6-fold increase in the level of the *brlf1* transcript was detected when BALF2, BMRF1 and BSLF1 were co-expressed with each of the three DNA binding domain ZEBRA mutants, Z(Y180E), Z(R187K) and Z(K188A). These results show that despite the defect in activating DNA lytic replication, all ZEBRA RD mutants were capable of activating transcription to levels equal to or higher than that of wt ZEBRA. In addition, replication proteins enhanced the capacity of wt ZEBRA and ZEBRA RD mutants to activate transcription of *brlf1*. This effect was more prominent when the BALF2-BMRF1-BSLF1 mixture was co-expressed with any of the three mutant forms of ZEBRA proteins containing single point mutations in the DNA recognition domain. The tripartite mixture of replication factors also enhanced the level of Rta protein activated by wild-type and mutant ZEBRA proteins. The enhancement was most marked for RD mutants Z(Y180E) and Z(R187K) (Fig. S4B).

## Discussion

In this study we provide evidence that a subset of virally encoded replication proteins enhance origin recognition by ZEBRA during lytic viral replication by promoting the capacity of ZEBRA to bind to viral DNA. ZEBRA binds specifically to a set of DNA sequences that are scattered throughout viral and cellular genomes. The EBV origin of lytic replication, oriLyt, is recognized by ZEBRA which also serves as a strong transcription activator by binding to lytic gene promoters. The ability of ZEBRA to perform two distinct functions in the same cell poses a biologically important question, namely, how would a protein like ZEBRA distinguish between a site that promotes transcription and another one that triggers replication? We addressed this question by characterizing a set of ZEBRA mutations that specifically disrupted the protein's ability to activate lytic viral replication (Fig. 1). These mutations are not significantly impaired at activating transcription and on most targets are enhanced as transcription activators (Fig. 2, 3, S4A and S5).

**Figure 11:**
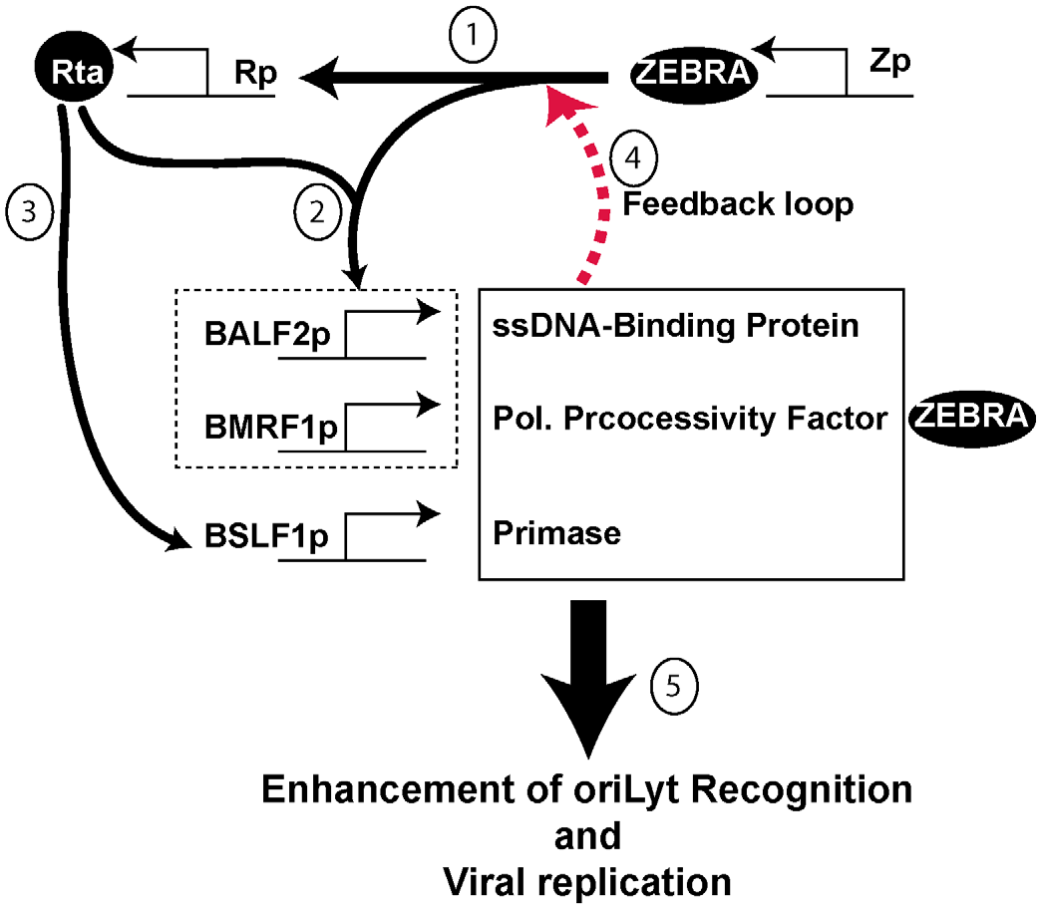
Proposed model for the role of BALF2, BMRF1 and BSLF1 in regulating viral replication. The model has several components: 1) ZEBRA activates Rta as a very early event of lytic cycle activation. 2) ZEBRA and Rta synergistically activate BALF2 and BMRF1. 3) Rta independently activates BSLF1 (data not shown). 4) The three replication proteins enhance the capacity of ZEBRA to activate transcription of Rta via a feedback loop (Fig. S4). 5) The three replication proteins enhance the interaction between ZEBRA and oriLyt (Fig. 9 and S2A).

A common defect observed among all five mutants was reduced DNA binding activity. Impairment of the ZEBRA mutants to interact with DNA was not specific to a particular ZEBRA response element and was detected whether we studied binding of ZEBRA to oriLyt or to promoters that regulate expression Rta, ZEBRA and BMRF1 (Fig. 5, 7, S2B and Table S1). However, the defect in DNA binding seemed to specifically disrupt activation of viral replication without affecting transcription (Fig. S5). This feature of the ZEBRA RD mutants allowed us to investigate the effect of EBV replication proteins on the interaction between ZEBRA and oriLyt and to correlate this effect with activation of viral replication. Increasing the concentration of all EBV replication proteins rescued the defect in viral replication and enhanced the formation of the ZEBRA-oriLyt complex (Fig. 8 and 9). A similar effect for replication proteins was observed with wt ZEBRA suggesting that this effect is not an artifact caused by the mutations installed in ZEBRA or due to failure of the RD mutants to activate expression of replication proteins. Subtraction experiments indicated that removal of the DNA polymerase (BALF5) from the mixture of replication proteins enhanced DNA replication while removal of expression vector encoding the viral primase (BSLF1) or the polymerase processivity factor (BMRF1) was detrimental (Fig. 10 and Fig. S1). In a reconstruction experiment, three EBV replication proteins were found to be sufficient to suppress the defect in replication and DNA binding associated with the ZEBRA RD mutants; these are the primase, the single-stranded DNA binding protein and the DNA polymerase processivity factor (Fig. 10 and Fig. S3). Expression of this tripartite replication mixture increased the level of Rta (*brlf1*) transcript and protein (Fig. S4). Thus, replication proteins seem to co-stimulate the capacity of ZEBRA to activate expression of Rta and consequently expression of replication factors prior to viral genome amplification. In summary, our findings support a model (Fig. 11) in which replication proteins promote lytic viral DNA synthesis in at least three different ways: i) replication proteins co-stimulate the capacity of ZEBRA to express Rta and other early lytic cycle products; ii) replication proteins augment the ability of ZEBRA to interact tightly with oriLyt, and iii) replication proteins comprise the EBV lytic replication machinery.

### Strong DNA binding is a requirement for viral replication

ZEBRA RD mutants with compromised DNA binding activity can be divided into two subclasses: the phosphorylation site mutants, Z(S173A) and Z(S167A/S173A), and the basic domain mutants, Z(Y180E), Z(R187K) and Z(K188A) [25], [28]. The defect in DNA binding was demonstrated using three different DNA binding assays: EMSA, ChIP and *in vivo* biotin-conjugated DNA affinity assay (iBDAA). Each of these assays addressed a different aspect of the DNA binding activity of ZEBRA. EMSA compared the capacity of the ZEBRA RD mutants to bind to individual ZREs *in vitro*. Four ZREs were tested, three present in oriLyt (ZRE1–3) and a fourth (ZIIIB) in Zp. The defect in binding to these sites by the ZEBRA RD mutants was severe relative to the wild type protein. However, examining the ability of the mutants to associate with oriLyt *in vivo* using ChIP revealed a milder defect (2- to 8-fold) (Fig. 6). This difference could be attributed to several factors; for example, ZEBRA binds to ZREs present in oriLyt in a cooperative manner [39], other viral proteins affect ZEBRA association to oriLyt (Fig. 9), and formation of pre-replication foci increases the local concentration of ZEBRA [40]. To directly assess the level of ZEBRA protein bound to oriLyt or Rp, we examined interaction of ZEBRA with biotinylated probes in BZKO cells. Using this in vivo biotinylated DNA affinity assay (iBDAA), we showed that all the mutants were impaired in their capacity to associate with both the oriLyt and Rp probes (Fig. 7).

Our studies on the DNA binding activity of ZEBRA revealed important correlations between strong interaction of ZEBRA with oriLyt and its capacity to activate viral replication. These correlations are: 1) all five ZEBRA mutants defective in activating viral replication exhibited a 2- to 8-fold defect in interacting with oriLyt (Fig. S2A). 2) The level of reduction in the DNA binding of each mutant correlated with its defect in stimulating viral replication (Fig. S5). 3) Replication proteins that enhanced interaction of ZEBRA with oriLyt restored viral replication (Fig. 9 and S2A). 4) At position S173, a mutation that disrupts DNA binding, e.g. Z(S173A), also abolishes viral replication, while another substitution that maintains DNA binding, e.g. Z(S173D), has no effect on viral replication [25]. All together these correlations point to the importance of strong interaction between ZEBRA and oriLyt to stimulate viral replication.

### Replication proteins play a role in origin recognition

Specific DNA binding by a protein that regulates different processes is not sufficient to confer specificity; additional levels of regulation likely play an important role beyond the initial step of DNA recognition. Consistent with the notion that initial recognition of the origin by the origin binding protein *per se* is not sufficient to induce replication, in *Saccharomyces cervisiae*, interaction with Cdc6p increased sequence-specific binding of ORC to the origin by altering its structure [29]. Also, the herpes simplex virus polymerase processivity factor (UL42) facilitated loading of the origin binding protein (UL9) to single-stranded or partially duplex DNA [41]. This study was done *in vitro* and the effect of replication proteins on the process of replication was not directly assessed in infected cells.

Here, we showed that expression of replication proteins enhanced interaction of ZEBRA with both oriLyt and Rp. This enhancement in binding is likely to have more impact on replication than on transcription of *brlf1* for the following reasons: 1) the ZEBRA RD mutants were fully competent to activate transcription of Rta and other lytic products. 2) Replication, and not transcription, was dependent on the capacity of ZEBRA to strongly bind to the corresponding viral DNA regulatory sites. 3) The replication proteins not only enhanced oriLyt recognition by the ZEBRA RD mutants but also restored their capacity to activate viral replication and late gene expression.

Over-expression of the tripartite mixture of replication factors did not rescue viral replication by the ZEBRA RD mutants to wild type level. This could be due to the presence of additional defects, other than DNA binding, in the ZEBRA RD mutants. Alternatively, over-expression of other viral or cellular proteins might be necessary to completely suppress the phenotype of these mutants in replication. However, a complete rescue of the mutants might be technically challenging since it is unlikely that all the cells will obtain and express the transfected plasmids.

Two findings suggest that replication proteins exert their effects early, during the assembly of the pre-replication complex or in the initial stages of replication rather than in extension. First, omission of the viral DNA polymerase (BALF5) expression vector markedly enhanced viral replication (Fig. S1). Second, addition of phosphonoacetic acid (PAA), an inhibitor of the viral DNA polymerase, had no effect on the ability of replication proteins to enhance ZEBRA association with oriLyt (Fig. 9). This effect of replication proteins is specific to three of the six replication proteins and is unlikely to be due to over-expression. In other EBV-infected cell lines, such as the Burkitt lymphoma derived cell line HH514-16, replication proteins are expressed at much higher levels than in transfected BZKO cells (Fig. 3A and [25]).

Origin recognition is a complex process that is regulated at multiple levels. In addition to the role of replication proteins in enhancing association of ZEBRA with oriLyt, other mechanisms must also be involved. For example, interaction of BBLF2/3 with ZBRK1 serves as a tethering point on oriLyt for other replication proteins [19]. The involvement of multiple mechanisms in regulation of origin recognition reflects the complexity of such an initial but essential step for activation of EBV replication.

### Replication proteins stimulate expression of Rta

At the initial stage of the EBV lytic cycle, stimulation of Rta expression by ZEBRA is independent of the presence of any replication proteins. As the lytic cycle proceeds into the early phase, ZEBRA and Rta, solely or synergistically, activate transcription of genes encoding the different components of the replication machinery. Our data shows that expression of three replication proteins, BALF2, BMRF1 and BSLF1 positively modulate the capacity of ZEBRA to stimulate expression of Rta (Fig. 2, S2B and S4). The co-stimulatory effect of this subset of replication proteins on Rta expression is likely to be a secondary event that occurs later during the pre-replicative phase of the lytic cycle. Our findings suggest that replication proteins trigger a positive feedback mechanism prior to viral replication that increases the level of Rta and replication proteins (Fig. 11). Upsurge in expression of replication proteins is likely to play a significant role in origin recognition, assembly of the replication complex and the process of viral DNA synthesis.

Evidence supporting the possible role of replication proteins in a positive feedback loop comes from a recent report suggesting that the DNA polymerase processivity factor, BMRF1, enhances the capacity of ZEBRA to activate the BALF2 promoter [42]. BMRF1 has also been shown to modulate the ability of Sp1 and ZBP-89 to activate the early viral BHLF1 promoter and the cellular gastrin promoter [43], [44]. The mechanism responsible for the transcriptional co-activation function of BMRF1 is still unknown. It is possible that the effect of replication proteins in augmenting the capacity of ZEBRA to activate transcription is mediated by BMRF1 only.

### Models for the role of replication proteins in origin recognition

The following models might account for the role of replication proteins in origin recognition. First, ZEBRA interacts with sub-complex(es) containing the three replication proteins, the primase, the ssDNA-binding protein and the DNA polymerase processivity factor, off DNA. This interaction results in the formation of a high affinity quaternary origin recognition complex. Second, ZEBRA binds independently to oriLyt and interacts with replication proteins that are already tethered to oriLyt through other cellular transcription factors, e.g. Sp1 and ZBRK1 [18], [19], [23]. The formation of this network of protein-protein interactions with multiple contacts among replication proteins, ZEBRA and oriLyt is likely to have a synergistic effect on the stability of this protein-DNA complex and to facilitate recruitment of other replication proteins [45]. Third, formation of the ZEBRA-oriLyt complex results in a specific DNA-protein architecture that functions as a landing pad for the three replication proteins which in turn augment and stabilize the interaction between ZEBRA and oriLyt. One possible function for the three replication proteins is to enhance the capacity of ZEBRA to occupy all the ZREs present in oriLyt (Fig. 9D and Fig. 10C). ZEBRA-oriLyt complexes that are not recognized by these three proteins are likely to become unstable and will fail to assemble a functional replication complex. These models do not yet account for the precise role of individual proteins. For example, it is possible that only one of these proteins, such as the ssDNA-binding protein, alters the origin binding capacity of ZEBRA while the two other proteins are important in subsequent events.

Based on our results, we propose that a tripartite mixture of replication proteins plays a role in EBV lytic origin recognition. This is a novel role for replication proteins. Additional experiments will be necessary to investigate the mechanism(s) by which each of these three replication proteins modulate the binding activity of ZEBRA to oriLyt and other ZEBRA response elements and enhance viral replication and transcription.

## Materials and Methods

### Plasmids

The plasmids pHD1013/Z, pHD1013/Z(S173A), pHD1013/Z(R187K), pHD1013/Z(Y180E), pHD1013/Z(K188A), pHD1013/Z(K188R), pHD1013/Z(F193E) and pHD1013/Z(K194A) were prepared as described previously [28], [46]. Expression vectors for the viral open reading frames encoding BALF5, BBLF4, BBLF2/3 and BSLF1 were a kind gift from Dr. Diane Hayward [4]. The full length coding sequences for BALF2 and BMRF1 were amplified from EBV genomes purified from HH514-16 cells by PCR. The amplified fragments were cloned in pFLAG-CMV2 using *EcoR1* and *Xba1* restriction sites.

### Cell culture and transfection

BZKO cells were previously described [1]. HKB5/B5 cells represent an EBV negative subclone that was initially generated by hybridizing HEK293 cells with the EBV positive cell line HH514-16 [47]. All transient transfection experiments were performed in 25 cm^2^ flasks using 3 µg of ZEBRA expression vector and 2 µg of each construct encoding a replication protein. The DMRIE-C reagent (Invitrogen) was used for transfection according to the manufacturer's protocol.

### Immunoblotting, immunoprecipitation and ChIP

Immunoblotting was performed as previously described [28]. The following antibodies were used: anti-ZEBRA, anti-FR3 and anti-LR2 are polyclonal rabbit sera raised to TrpE-fusion proteins expressed in E. coli. The anti-Rta antibody was generated by expressing the C-terminal 320 a.a of Rta using the pET-expression system. The fragment was purified on a nickel column and used for rabbit immunization. The monoclonal antibody against BMRF1 (EA-D) (R3.1) was a kind gift from G. Pearson. Anti-FLAG is a mouse monoclonal antibody (Sigma). ChIP experiments were performed as previously detailed [25]. Sequences for the primers used are available upon request.

### Northern and Southern blot analyses

RNA was purified from 8×10^6^ BZKO or HH514-16 cells using RNeasy kits (Qiagen) according to the manufacturer's protocol. All RNA samples were treated with 30 U DNase1 (Qiagen). Twenty micrograms of RNA was separated on 1% agarose gel and transferred by capillarity to a Hybond N+ filter (Amersham). The membrane was hybridized to two ^32^P-labeled probes detecting the H1 component of RNase P (a loading control) and BALF2. The probes were generated from a 370-bp NcoI-Pst1 fragment of RNase P and full length BALF2 DNA using random primers.

DNA was isolated from 10^7^ BZKO cells as detailed previously [28]. Ten micrograms of DNA was digested with 40 units BamH1 (New England Biolabs) for 3 h at 37°C. DNA fragments were separated by electrophoresis in a 0.8% agarose gel and transferred to a Zeta probe GT genomic membrane (Bio-Rad). Formation of a replication ladder was detected using a probe complementary to a 336-bp sequence in the unique Xho 1.9-kb sequence upstream of the viral terminal repeats [34]. The template for the 336-bp probe was generated by PCR using the following primers 5′-CTCACGAGCAGGTGG-3′ and 5′-CGCAGTCTTAGGTATCTGG-3′. An excised EBV BamH1 W fragment was used as a template to generate a corresponding probe [48]. Radioactive probes were synthesized using 10 units of the Klenow fragment of DNA polymerase (New England Biolabs), [α-^32^P]dCTP and 1 ng random primers. The probes were purified using a Sephadex-G50 column.

### Reverse transcription and quantitative PCR

RNA samples were prepared 48 h after transfection of BZKO cells. Phosphonoacetic acid (PAA) was added to inhibit viral replication. RT-PCR was performed on 100 ng of total RNA using reagents and instructions described in the manual for the SuperScript One-Step RT-PCR with platinum Taq kit (Invitrogen). In reactions where the reverse transcriptase was omitted, 2 units of platinum Taq was added. Random hexamers or gene specific primers were used to generate cDNA. A 131-bp fragment was amplified by the BBLF4 primers; a 121-bp fragment by the BSLF1 primers, and 211-bp by the BBLF2/3 primers. The sequences for the primers used are available upon request. Incorporation of Sybr green into DNA was detected using Cepheid Smart Cycler II or a Bio-Rad MyiQ real time PCR machines. Standard curves were generated using 10-fold serial dilutions of expression vectors encoding each of the three open reading frames. Quantitative PCR for detection of viral genome amplification was previously described [25].

### EMSA

Preparation of supernatants of HKB5/B5 cell extracts expressing wt ZEBRA or mutants as well as the DNA binding reactions were previously described [28]. Super-shifts were performed using BZ1, a monoclonal antibody against the dimerization domain of ZEBRA. The percent probe shifted is calculated as previously described [28], [49].

### Binding to Biotin-labeled oriLyt

Full length oriLyt was cloned into pBSKII+ from HH514-16 cells using primers containing *EcoR1* and *BamH1* sites 5′-GCGCGAATTCTGGGGTCTCTGTGTAATACTTTAAG-3′ and 5′-GCGCGGATCCGTTA ATAAGGAGCC GTCCTTATTC-3′. Biotin-labeled full length oriLyt (BoF) was prepared by PCR using primers that were conjugated to biotin at their 5′ends. BZKO cells were co-transfected with 150 ng BoF and the indicated expression vectors. Cells were harvested after 60 h and re-suspended in lysis buffer containing 15 mM Tris-HCl pH 8.1, 167 mM NaCl, 1.2 mM EDTA, 3 mM MgCl_2_, 0.01% SDS and 1.1% Triton X-100. Cell extracts were briefly sonicated and supernatants were collected. The amounts of total protein were assessed using the Bradford reagent (Bio-Rad) and equalized. ZEBRA bound to BoF was captured using Avidin coated beads. The beads were washed and heated in SDS-PAGE sample buffer. The amount of captured ZEBRA protein was determined using Western blot analysis.

## Supporting Information

Figure S1 Effect of excluding individual components from the mixture of EBV replication proteins on stimulation of viral replication by Z(S173A). Quantitative-PCR was used to examine relative EBV genome amplification in BZKO cells. Cells were transfected with empty vector (CMV), wild type ZEBRA (Z) or the ZEBRA mutant Z(S173A). Where indicated, the two forms of ZEBRA were expressed with all six replication proteins (RPs). Alternatively, a single component of the replication machinery was omitted and the other five proteins were co-expressed with Z(S173A). After 48 h, the cells were harvested and the concentration of viral DNA was measured using primers specific to the upstream region of oriLyt. Relative genome amplification was calculated by comparison to DNA amplification by wt ZEBRA protein which was set at 100.
(0.21 MB TIF)

Figure S2 Replication proteins augment the association of ZEBRA with oriLyt and Rp. Compilation of data from multiple ChIp experiments examining the capacity of ZEBRA RD mutants to interact with oriLyt (A) or Rp (B) and the effect of replication proteins (RPs) on these interactions. Quantitative PCR data obtained from each ChIp experiment was initially corrected for the amount of input DNA and then normalized to the amount of oriLyt or Rp precipitated from cells transfected with empty vector. The extent of binding of each ZEBRA RD mutant to DNA was then normalized to DNA binding by wt ZEBRA in the absence of replication proteins. The letter n represents the number of biological replicates for each condition. If n was more than one, the average binding capacity of each mutant was calculated based on values obtained from biological replicates. Each real time PCR value used in this analysis was an average of three technical repeats.
(0.52 MB TIF)

Figure S3 Overexpression of BALF2, BMRF1 and BSLF1 partially restores the genome amplification defect of ZEBRA RD mutants. The indicated expression vectors were transfected into BZKO cells. MSA represents plasmids encoding BMRF1, BSLF1 and BALF2, respectively. After 48 h (panel A) and 72 h (panel B) the cells were harvested and DNA was purified. Quantitative PCR was performed to assess the extent of EBV genome amplification. Primers specific to the oriLyt region were used to measure the amount of viral DNA synthesized under each condition. The fold change in the level of viral DNA activated by each ZEBRA RD mutant in the absence and presence of the MSA mixture of replication proteins was calculated and compared to wt ZEBRA.
(0.51 MB TIF)

Figure S4 Replication proteins induce a co-stimulatory effect on expression of Rta. A) Quantitative PCR to determine changes in the level of brlf1 transcript following expression of the indicated forms of ZEBRA in the absence or presence of BALF2, BMRF1 and BSLF1. Viral replication was blocked by PAA. BZKO cells were harvested after 24 hours. The figure represents the average of two biological replicate experiments. B) Western blot analysis for the level of Rta protein induced by wt ZEBRA or the indicated ZEBRA mutants in the absence and presence of the tripartite mixture of replication proteins. MSA represents plasmids encoding BMRF1, BSLF1 and BALF2, respectively.
(0.49 MB TIF)

Figure S5 Comparison between the capacity of ZEBRA to bind to Rp and oriLyt with its ability to activate transcription of Rta and DNA replication. A compilation of several experiments already presented in the manuscript. Data representing activation of the brlf1 (Rta) transcript is the average of three experiments presented in Fig. 2 and S4. Association of ZEBRA with Rp or oriLyt was presented in Fig. S2A and 2B, respectively. Quantitative PCR determining the extent of viral genome amplification was acquired from Fig. S3A. Together the data demonstrates that the defect in DNA binding associated with the ZEBRA RD mutants has no effect on transcription but has adverse effects on replication.
(1.05 MB TIF)

Table S1 Summary of chromatin immunoprecipitation experiments
(0.03 MB DOC)
