# Supplementary figures and images for: A Subset of Replication Proteins Enhances Origin Recognition and Lytic Replication by the Epstein-Barr Virus ZEBRA Protein
Source: PLoS Pathog. 2010 Aug 19;6(8):e1001054. doi: 10.1371/journal.ppat.1001054 (PMC2924361; doi:10.1371/journal.ppat.1001054)

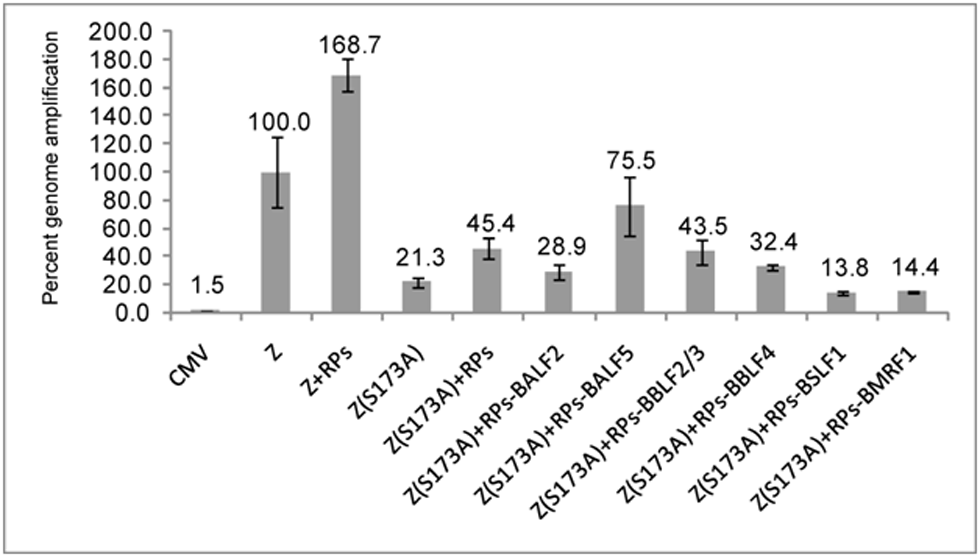

Supplement: Figure S1 — Effect of excluding individual components from the mixture of EBV replication proteins on stimulation of viral replication by Z(S173A). Quantitative-PCR was used to examine relative EBV genome amplification in BZKO cells. Cells were transfected with empty vector (CMV), wild type ZEBRA (Z) or the ZEBRA mutant Z(S173A). Where indicated, the two forms of ZEBRA were expressed with all six replication proteins (RPs). Alternatively, a single component of the replication machinery was omitted and the other five proteins were co-expressed with Z(S173A). After 48 h, the cells were harvested and the concentration of viral DNA was measured using primers specific to the upstream region of oriLyt. Relative genome amplification was calculated by comparison to DNA amplification by wt ZEBRA protein which was set at 100. (0.21 MB TIF) [file ppat.1001054.s001.tif]

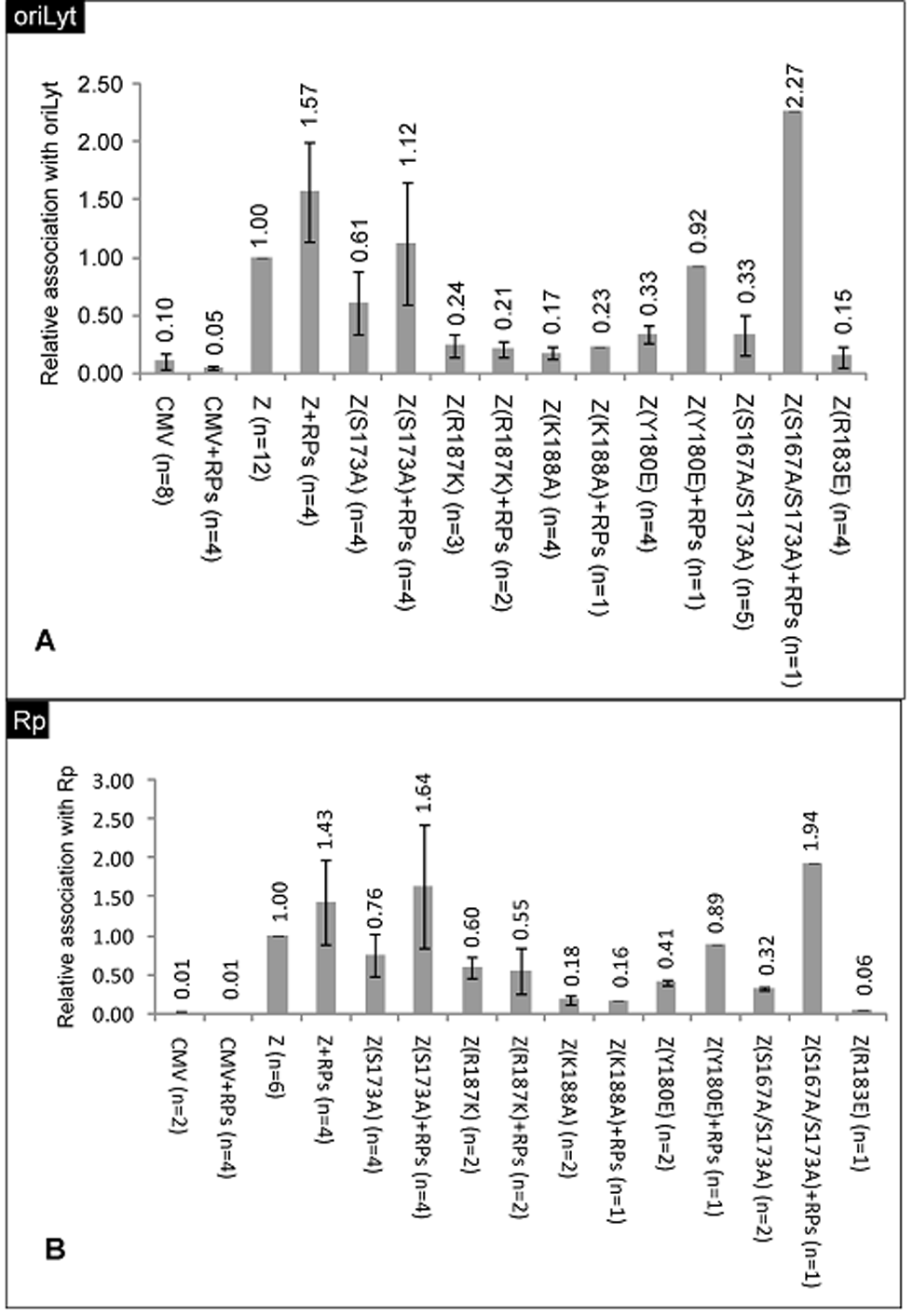

Supplement: Figure S2 — Replication proteins augment the association of ZEBRA with oriLyt and Rp. Compilation of data from multiple ChIp experiments examining the capacity of ZEBRA RD mutants to interact with oriLyt (A) or Rp (B) and the effect of replication proteins (RPs) on these interactions. Quantitative PCR data obtained from each ChIp experiment was initially corrected for the amount of input DNA and then normalized to the amount of oriLyt or Rp precipitated from cells transfected with empty vector. The extent of binding of each ZEBRA RD mutant to DNA was then normalized to DNA binding by wt ZEBRA in the absence of replication proteins. The letter n represents the number of biological replicates for each condition. If n was more than one, the average binding capacity of each mutant was calculated based on values obtained from biological replicates. Each real time PCR value used in this analysis was an average of three technical repeats. (0.52 MB TIF) [file ppat.1001054.s002.tif]

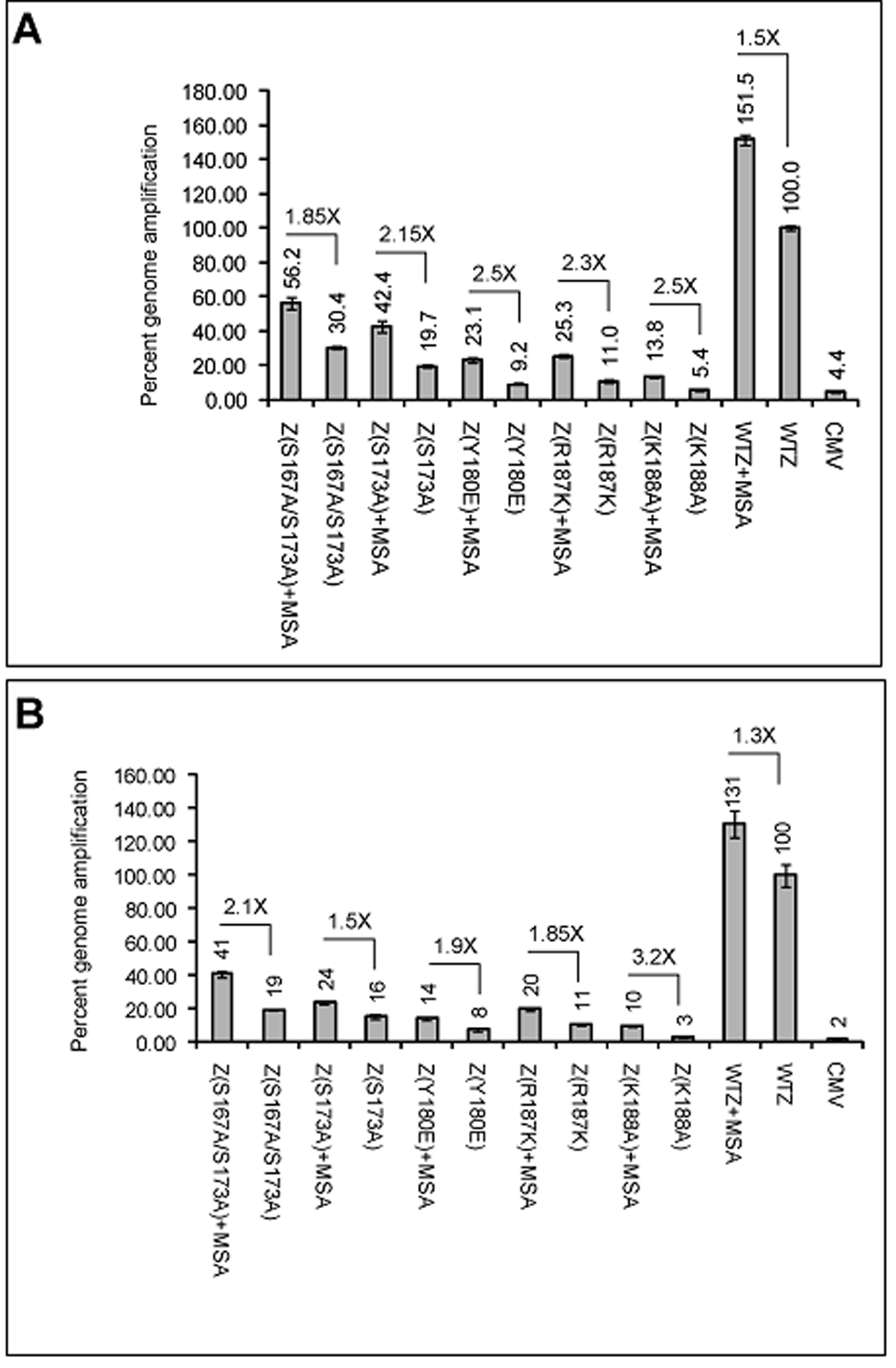

Supplement: Figure S3 — Overexpression of BALF2, BMRF1 and BSLF1 partially restores the genome amplification defect of ZEBRA RD mutants. The indicated expression vectors were transfected into BZKO cells. MSA represents plasmids encoding BMRF1, BSLF1 and BALF2, respectively. After 48 h (panel A) and 72 h (panel B) the cells were harvested and DNA was purified. Quantitative PCR was performed to assess the extent of EBV genome amplification. Primers specific to the oriLyt region were used to measure the amount of viral DNA synthesized under each condition. The fold change in the level of viral DNA activated by each ZEBRA RD mutant in the absence and presence of the MSA mixture of replication proteins was calculated and compared to wt ZEBRA. (0.51 MB TIF) [file ppat.1001054.s003.tif]

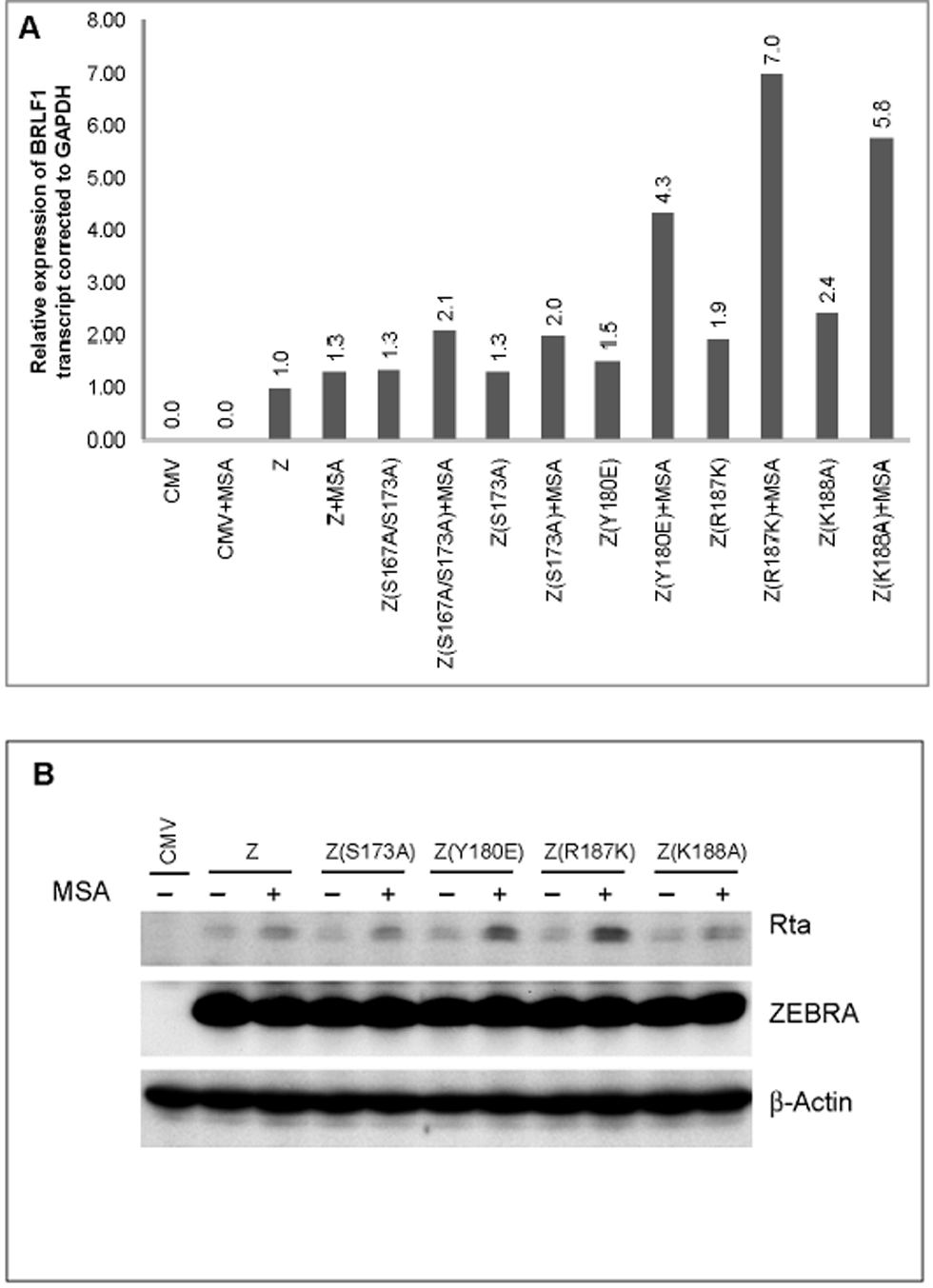

Supplement: Figure S4 — Replication proteins induce a co-stimulatory effect on expression of Rta. A) Quantitative PCR to determine changes in the level of brlf1 transcript following expression of the indicated forms of ZEBRA in the absence or presence of BALF2, BMRF1 and BSLF1. Viral replication was blocked by PAA. BZKO cells were harvested after 24 hours. The figure represents the average of two biological replicate experiments. B) Western blot analysis for the level of Rta protein induced by wt ZEBRA or the indicated ZEBRA mutants in the absence and presence of the tripartite mixture of replication proteins. MSA represents plasmids encoding BMRF1, BSLF1 and BALF2, respectively. (0.49 MB TIF) [file ppat.1001054.s004.tif]

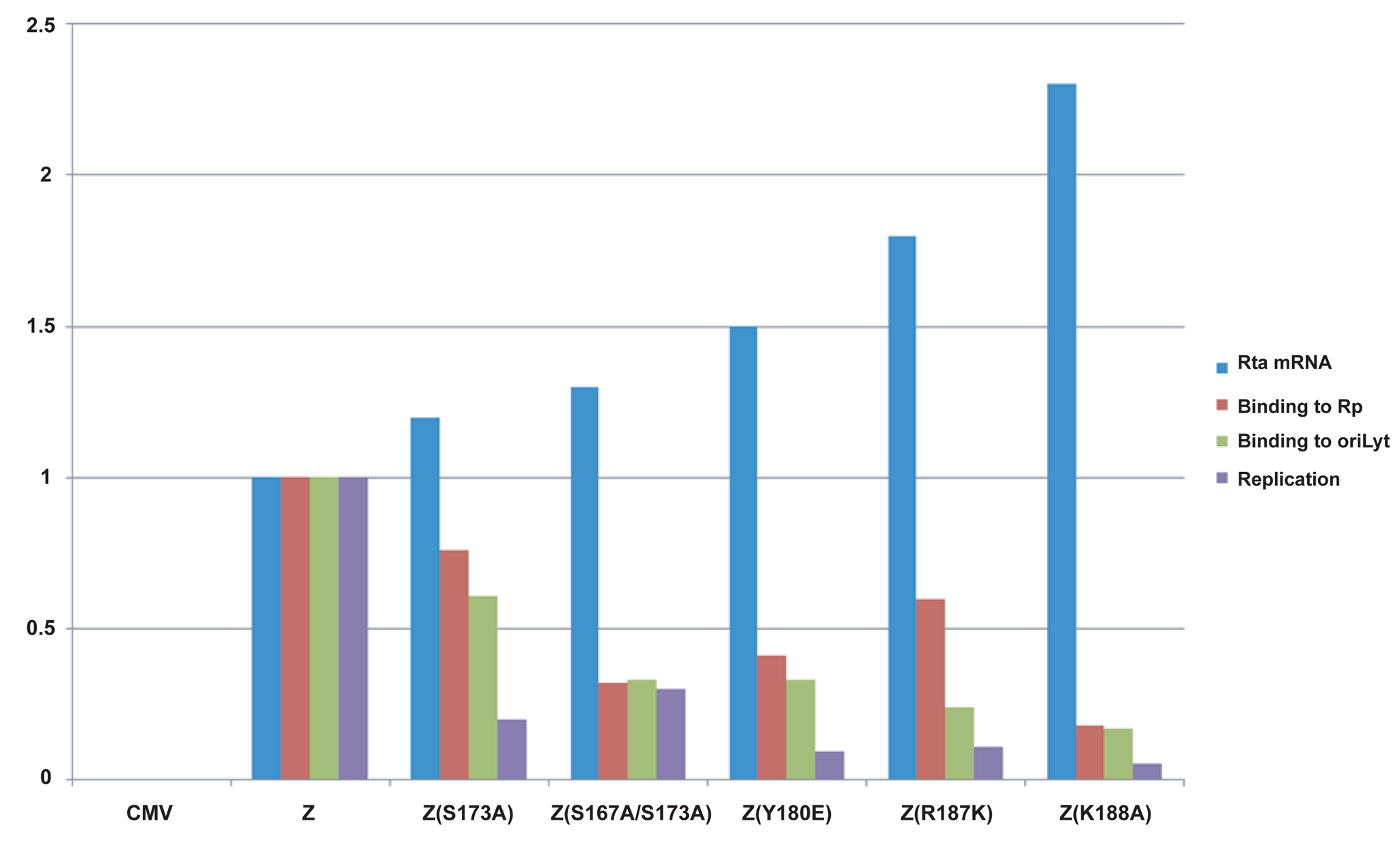

Supplement: Figure S5 — Comparison between the capacity of ZEBRA to bind to Rp and oriLyt with its ability to activate transcription of Rta and DNA replication. A compilation of several experiments already presented in the manuscript. Data representing activation of the brlf1 (Rta) transcript is the average of three experiments presented in Fig. 2 and S4. Association of ZEBRA with Rp or oriLyt was presented in Fig. S2A and 2B, respectively. Quantitative PCR determining the extent of viral genome amplification was acquired from Fig. S3A. Together the data demonstrates that the defect in DNA binding associated with the ZEBRA RD mutants has no effect on transcription but has adverse effects on replication. (1.05 MB TIF) [file ppat.1001054.s005.tif]
