# Supplementary material for: A Subset of Replication Proteins Enhances Origin Recognition and Lytic Replication by the Epstein-Barr Virus ZEBRA Protein
Source: PLoS Pathog. 2010 Aug 19;6(8):e1001054. doi: 10.1371/journal.ppat.1001054 (PMC2924361; doi:10.1371/journal.ppat.1001054)
Supplement: Table S1 — Summary of chromatin immunoprecipitation experiments (0.03 MB DOC) [file ppat.1001054.s006.doc]

Supplemental Table 1. Summary of chromatin immunoprecipitation experiments.

|  | | | | | | | | |
| --- | --- | --- | --- | --- | --- | --- | --- | --- |
|  | oriLyt | | Rp | | Zp | | BMRF1p | |
|  | -RPs | +RPs | -RPs | +RPs | -RPs | +RPs | -RPs | +RPs |
| Wt Z | 1 | 1.57 | 1 | 1.43 | 1 | 1.79 | 1 | 2.2 |
| Z(S167A/S173A) | 0.33 | 2.27 | 0.32 | 1.94 | 0.45 | 1.39 | 0.9 | 2.2 |
| Z(S173A) | 0.61 | 1.12 | 0.7 | 1.64 | 0.61 | 2.02 | 0.7 | 2.1 |
| Z(Y180E) | 0.33 | 0.92 | 0.41 | 0.89 | 0.39 | 0.52 | 0.4 | 0.5 |
| Z(R187K | 0.24 | 0.21 | 0.6 | 0.55 |  |  |  |  |
| Z(K188A) | 0.17 | 0.23 | 0.18 | 0.16 |  |  |  |  |
